# Apolipoprotein (a)/Lipoprotein(a)-Induced Oxidative-Inflammatory *α*7-nAChR/p38 MAPK/IL-6/RhoA-GTP Signaling Axis and M1 Macrophage Polarization Modulate Inflammation-Associated Development of Coronary Artery Spasm

**DOI:** 10.1155/2022/9964689

**Published:** 2022-01-19

**Authors:** Yen-Kuang Lin, Chi-Tai Yeh, Kuang-Tai Kuo, Iat-Hang Fong, Vijesh Kumar Yadav, Nicholas G. Kounis, Patrick Hu, Ming-Yow Hung

**Affiliations:** ^1^Graduate Institute of Athletics and Coaching Science, National Taiwan Sport University, Taoyuan 33301, Taiwan; ^2^Biostatistics Research Center, Taipei Medical University, Taipei, Taiwan; ^3^Department of Medical Research and Education, Taipei Medical University-Shuang Ho Hospital, New Taipei City, Taiwan; ^4^Department of Hematology and Oncology, Taipei Medical University-Shuang Ho Hospital, New Taipei City, Taiwan; ^5^Division of Thoracic Surgery, Department of Surgery, Shuang Ho Hospital, Taipei Medical University, New Taipei City, Taiwan; ^6^Division of Thoracic Surgery, Department of Surgery, School of Medicine, College of Medicine, Taipei Medical University, Taipei, Taiwan; ^7^Division of Cardiology, Department of Internal Medicine, University of Patras Medical School, 26221 Patras, Greece; ^8^University of California, Riverside, California, USA; ^9^Department of Cardiology, Riverside Medical Clinic, Riverside, California, USA; ^10^Division of Cardiology, Department of Internal Medicine, School of Medicine, College of Medicine, Taipei Medical University, Taipei, Taiwan; ^11^Division of Cardiology, Department of Internal Medicine, Shuang Ho Hospital, Taipei Medical University, New Taipei City, Taiwan; ^12^Taipei Heart Institute, Taipei Medical University, Taipei, Taiwan

## Abstract

**Objective:**

Apolipoprotein (a)/lipoprotein(a) (Lp(a)), a major carrier of oxidized phospholipids, and *α*7-nicotinic acetylcholine receptor (*α*7-nAChR) may play an important role in the development of coronary artery spasm (CAS). In CAS, the association between Lp(a) and the *α*7-nAChR-modulated inflammatory macrophage polarization and activation and smooth muscle cell dysfunction remains unknown.

**Methods:**

We investigated the relevance of Lp(a)/*α*7-nAChR signaling in patient monocyte-derived macrophages and human coronary artery smooth muscle cells (HCASMCs) using expression profile correlation analyses, fluorescence-assisted cell sorting flow cytometry, immunoblotting, quantitative real-time polymerase chain reaction, and clinicopathological analyses.

**Results:**

There are increased serum Lp(a) levels (3.98-fold, *p* = 0.011) and macrophage population (3.30-fold, *p* = 0.013) in patients with CAS compared with patients without CAS. Serum Lp(a) level was positively correlated with high-sensitivity C-reactive protein (*r*^2^ = 0.48, *p* < 0.01), IL-6 (*r*^2^ = 0.38, *p* = 0.03), and *α*7-nAChR (*r*^2^ = 0.45, *p* < 0.01) in patients with CAS, but not in patients without CAS. Compared with untreated or low-density lipoprotein- (LDL-) treated macrophages, Lp(a)-treated macrophages exhibited markedly enhanced *α*7-nAChR mRNA expression (*p* < 0.01) and activity (*p* < 0.01), *in vitro* and *ex vivo*. Lp(a) but not LDL preferentially induced CD80+ macrophage (M1) polarization and reduced the inducible nitric oxide synthase expression and the subsequent NO production. While shRNA-mediated loss of *α*7-nAChR function reduced the Lp(a)-induced CD80+ macrophage pool, both shRNA and anti-IL-6 receptor tocilizumab suppressed Lp(a)-upregulated *α*7-nAChR, p-p38 MAPK, IL-6, and RhoA-GTP protein expression levels in cultures of patient monocyte-derived macrophages and HCASMCs.

**Conclusions:**

Elevated Lp(a) levels upregulate *α*7-nAChR/IL-6/p38 MAPK signaling in macrophages of CAS patients and HCASMC, suggesting that Lp(a)-triggered inflammation mediates CAS through *α*7-nAChR/p38 MAPK/IL-6/RhoA-GTP signaling induction, macrophage M1 polarization, and HCASMC activation.

## 1. Introduction

Studies in patients with coronary artery spasm (CAS), an intense coronary vasoconstriction, have substantially contributed to the understanding of myocardial ischemia [1–3]. CAS is an inflammatory disease characterized by elevated peripheral monocyte count [4], plasma levels of high-sensitivity C-reactive protein (hs-CRP) [5, 6], and interleukin- (IL-) 6 [7], while enhanced serum IL-6 and hs-CRP levels attenuate endothelial nitric oxide (NO) synthase activity and suppress NO production [8–10], leading to CAS development. IL-6, a primary determinant of hepatic production of CRP, not only contributes to the inflammatory response but also has been shown to be associated with endothelial dysfunction and consequently plays an important role in CAS [2].

Cholinergic signaling and nicotinic acetylcholine receptors (nAChRs) have recently gained focus in cardiovascular morbidity and mortality [11, 12]. The *α*7-nAChRs, encoded by the CHRNA7 gene, are of relevance in inflammation [9] and expressed by mononuclear inflammatory cells, including monocytes and monocyte-derived macrophages [13, 14]. We previously demonstrated that the activation of monocytic *α*7-nAChR exacerbated oxidative stress and promoted CAS through a p38 mitogen-activated protein kinase- (MAPK-) dependent mechanism [9], which is in line with the studies of the nicotinic atherogenic effects of nAChR [11, 12]. Furthermore, the excessive vascular smooth muscle cell (VSMC) contraction in CAS has been related to Ras-homologous (Rho) A GTPase/Rho-kinase (ROCK1, ROCK2) pathway, which can induce inflammation and oxidative stress [15, 16]. Notably, *α*7-nAChR is involved in the activation of the Rho GTPase pathway and the downstream signaling pathway in VSMCs [17], which may lead to CAS. Despite the important role of *α*7-nAChR in CAS, the development of molecular modulators of *α*7-nAChR and the associated therapeutic translational research in CAS has remained largely unknown [18]. Therefore, we further explored probable druggable molecular modulators of *α*7-nAChR underlying CAS development.

On the other hand, lipoprotein(a) (Lp(a)), a major carrier of oxidized phospholipids, has been observed to play an important role in CAS development and related myocardial infarction [19, 20]. Moreover, elevated Lp(a) level is a causal risk factor for coronary artery disease (CAD) and may similarly play an important role in other atherothrombotic disorders [21]. While atherogenic lipoproteins significantly modulate vascular tone, oxidized Lp(a) is more potent than oxidized LDL [22]. The disproportionately large impact of Lp(a) on cardiovascular disease risk compared with low-density lipoprotein implies that additional pathogenic pathways need to be considered. Moreover, despite the implication of *α*7-nAChR and Lp(a) in CAS and shared molecular mediators [7–9, 20] between them, it remains undetermined whether CAS is due to the 2 conditions sharing common inflammatory factors or whether shared inflammatory factors provide the link. There is increasing evidence that blood monocyte function may be changed by dyslipidemia [23]. Hence, the present study examined probable interaction and modulatory loop between Lp(a) and monocytic *α*7-nAChR in patients with CAS.

The agonists of the nAChR include nicotine, epibatidine, choline [24], and the endogenous agonist acetylcholine, which has been used to provoke and diagnose CAS during coronary angiography [3]. Because nicotine is the major reinforcing component and psychoactive drug of tobacco smoke [25], both nicotine in the tobacco smoke [26] and endogenous acetylcholine may contribute to the CAS development. We previously demonstrated that activation of the monocytic *α*7-nAChRs modulates oxidative stress and inflammation-associated development of CAS via a p38 MAP-kinase signaling-dependent pathway [9]. In addition, positive interactions exist among CRP, hemoglobin, and platelet in women with CAS, but not in men [27]. While hemoglobin is a modifier in CAS development in women, platelet count is an independent risk factor for men [27]. Because hemoglobin levels and platelet counts have been found to vary substantially according to age, gender, and race/ethnicity [28, 29], population-based studies are needed for hemoglobin and platelet to differentiate the causality from predisposing factors through biomarkers to the occurrence of CAS. On the other hand, the potential role for platelet-released factors in CAS would not necessarily imply an abnormality in platelet function [30]. The primary abnormality might be increased sensitivity of VSMCs to normal levels of vasoconstrictive agents such as thromboxane A2 [30]. Furthermore, CAS could be the primary event and the CAS-induced “stasis” in the coronary artery might lead to an increase in the numbers of circulating platelet aggregates, suggesting a potential causal role of platelets in CAS [30]. Loscalzo et al. have demonstrated that intravenous administration of nitroglycerin inhibits cyclic blood flow responses caused by periodic platelet thrombus formation in stenosed canine coronary arteries [31], and N-acetylcysteine markedly potentiates the inhibition of platelet aggregation by nitroglycerin [32]. While platelet resistance to NO is aggravated during acute symptomatic CAS episodes, mast cell activation and damage to both vasculature and platelets also occur [33]. N-Acetylcysteine, via release of H2S, reverses platelet resistance to NO and terminates glycocalyx shedding during symptomatic CAS crises, suggesting that H2S donors may correct the pathophysiological anomalies [33]. Forman et al. [34] revealed that more mast cells were found in the adventitia of the involved artery in patients with CAS than in patients with CAD and sudden death but without CAS or in normal controls who died in accidents, which raised the possibility that products derived from mast cells (histamine, prostaglandin D2, and leukotrienes C4 and D4) may partly mediate CAS in some patients. These important studies suggest that the loss of NO effect predisposes coronary vessels towards microthrombosis, endothelial damage, and ongoing inflammation. Notably, Kounis syndrome is defined as the coexistence of acute coronary syndromes including CAS, acute myocardial infarction, and stent thrombosis, with allergic or hypersensitivity conditions associated with mast cell and platelet activation [35]. Collectively, although endothelial cell dysfunction might favor the induction of CAS, other factors may also be involved in the pathogenesis of CAS. On the other hand, the involvement of the *α*7-nAChR in the development of atherosclerosis is yet an expanding field, as both atheroprotective and proatherogenic roles are attributed to the stimulation of *α*7nAChRs, and their role in the genesis and progression of atheromatous plaque is still under debate. In vivo studies revealed both anti- and proatherogenic effects [36]. In vitro studies indicated that the activation of *α*7-nAChRs regulates the function of different cells involved in a variety of pathways linked to plaque progression [36]. Stimulation of vascular *α*7-nAChRs contributes to angiogenesis and proliferation of VSMCs and may promote atherogenesis [36]. High Lp(a) concentrations (>50 mg/dL) are associated with significantly increased risk of myocardial infarction in all populations except Arabs and Africans [37]; however, the relationship between Lp(a) and *α*7-nAChR remains largely unknown. We recently demonstrated that garcinol attenuates Lp(a)-induced oxidative stress and inflammatory cytokine production in ventricular cardiomyocyte through *α*7-nAChR-mediated inhibition of the p38 MAPK and NF-*κ*B signaling pathways in a mouse model of myocardial infarction [38], suggesting an important role of *α*7-nAChR and its downstream signaling mechanisms in regulating Lp(a)-induced cardiomyocyte apoptosis and inflammation. In addition, CAS is considered as one of the causes of acute coronary syndrome with plaque rupture [39] or myocardial infarction with nonobstructive coronary artery [40]. Therefore, while scarce data are available on Lp(a) in relation to *α*7-nAChR and CAS, more studies are warranted before Lp(a)/*α*7-nAChR-mediated responses could be considered as a therapeutic target for CAS.

Clinically, CAS is characterized by transient myocardial ischemia followed by reperfusion [41]. Repeat ischemia-reperfusion can stimulate proinflammatory responses from coronary VSMCs [42], which may increase the risk of developing CAS [43]. As part of the pathogenesis of atherosclerosis, inflammatory signals stimulate proinflammatory responses from macrophages and VSMCs [44], which in turn may exacerbate CAS. Taken together, these observations suggest a role for coronary VSMC-related inflammation in CAS development. On the other hand, Lp(a) acts in a species-specific manner on cultured rat and human VSMCs [45]. To date, no information is available concerning the effects of Lp(a) on monocyte-derived macrophages in patients with CAS and human coronary artery smooth muscle cells (HCASMCs). We, therefore, analyzed the protein expression levels of Lp(a) and *α*7-nAChR in the monocytes of patients with CAS. Furthermore, we investigated the effects of Lp(a) on monocyte-to-macrophage differentiation and polarization based on CD80 or CD206 positivity and *α*7-nAChR-dependent activation of the p38 MAPK signaling in monocyte-derived macrophages and primary HCASMCs.

## 2. Material and Methods

### 2.1. Cells, Compounds, and Reagents

The primary HCASMCs (ATCC® PCS-100-021™, American Type Culture Collection, Manassas, VA, USA) were cultured in smooth muscle cell growth medium 2 (#C-22062, PromoCell GmbH, Heidelberg, Germany), and all patient monocyte-derived macrophage cells were cultured in the RPMI-1640 culture media (Sigma-Aldrich Corporation, St. Louis, MO, USA) supplemented with 10% fetal bovine serum (FBS), 2 mM glutamine, 50 *μ*g/mL streptomycin, and 50 U/mL penicillin in 5% CO_2_ humidified atmosphere incubator at 37°C to 98%-100% confluence. Cells were subcultured and culture media changed every 48 h. Human CRP (#C1617, Sigma-Aldrich Corporation, St. Louis, MO, USA), human IL-6 (#407652, purity ≥ 95% by SDA-PAGE, Sigma-Aldrich Corporation, St. Louis, MO, USA), human low-density lipoprotein (LDL) (#LP2, purity ≥ 95% by SDA-PAGE, Sigma-Aldrich Corporation, St. Louis, MO, USA), methyllycaconitine (MLA), a selective and potent antagonist of the *α*7-nAChR, (351344-10-0, caymanchem, USA), and anti-IL-6 receptor antibody (tocilizumab) were also obtained from Sigma-Aldrich. Stock solutions of tocilizumab were prepared at a concentration of 10 mM in double-distilled water (ddH_2_O) and stored at -20 °C until use. Methylergonovine (Methergine®) was obtained from Novartis (Novartis Pharmaceuticals Corp., Basel, Switzerland) and nitroglycerin from G. Pohl-Boskamp (Millisrol®; G. Pohl-Boskamp, Hohenlockstedt, Germany).

### 2.2. Study Population

This prospective cohort study was carried out with the approval of the Taipei Medical University Joint Institutional Review Board (approval number: TMU-JIRB N201903036). All patients provided signed informed consent regarding use of their blood in scientific research, and the study was compliant with the guidance of the Declaration of Helsinki for biomedical research involving human subjects. A total of 64 patients (45 men and 19 women), who had chest pain and suspected ischemic heart disease on noninvasive tests, undergoing diagnostic coronary angiography with or without established CAS, but without obstructive stenosis, from July 2017 to March 2019 were enrolled in this study. Study subjects were stratified into control (*n* = 32) and CAS (*n* = 32) groups. Among them, 9 from the control and 10 CAS subjects were active smokers. Inclusion criteria for patients with CAS included spontaneous chest pain at rest associated with ST-segment elevation or depression on electrocardiogram that was relieved by sublingual administration of nitroglycerin, no angiographic evidence of obstructive CAD after intracoronary nitroglycerin administration, and a positive result on intracoronary methylergonovine provocation testing. CAS was not induced in the remaining 32 patients (non-CAS, control), which consisted of patients who presented with atypical chest pain, no angiographic evidence of obstructive CAD, and negative results on intracoronary methylergonovine provocation testing (no CAS). Atypical chest pain was defined as spontaneous chest pain at rest and/or provoked by exertion that was eased by sublingual administration of nitroglycerin [46] but not linked with ST-segment change on resting electrocardiogram. Exclusion criteria included the presence of obstructive CAD, coronary microvascular spasm [47], inflammatory manifestations probably associated with noncardiac diseases (e.g., infections and autoimmune disorders), liver disease/renal failure (serum creatinine level > 2.5 mg/dL), collagen disease, malignancy, and loss of blood samples. None of our patients had allergic or hypersensitivity conditions.

### 2.3. Data Collection

For this study, patients' demographic, anthropometric, and laboratory data as well as details of their comorbidities, medicine use, habits, and number of functional units were collected. Current smoking was defined as having smoked a cigarette within 3 weeks of cardiac catheterization. Diabetes mellitus was diagnosed when the fasting glucose level was ≥126 mg/dL on >2 occasions or was defined from dietary treatment and/or medical therapy. Baseline seated blood pressure was derived from the mean of 6 readings obtained during the first 2 office visits at 2 weeks apart. Hypertension was defined as a blood pressure > 140/90 mmHg on >2 occasions or receiving antihypertensive treatment.

### 2.4. Spasm Provocation Test Protocol

The standard Judkins technique was employed for coronary angiography [8]. Nitrates and calcium antagonists were withdrawn for ≥24 h before the procedure. Left ventricular ejection fraction was calculated using Simpson's method. Obstructive CAD was defined as a ≥50% decrease in luminal diameter after administration of intracoronary nitroglycerin [9]. If no obstructive CAD was discovered, intracoronary methylergonovine (Methergine®; Novartis, Basel, Switzerland) was given stepwise (1, 5, 10, and 30 *μ*g) first into the right coronary artery and subsequently into the left coronary artery. CAS was defined as a >70% reduction in luminal diameter compared with postintracoronary nitroglycerin, with associated angina and/or ST depression or elevation [9]. Provocation testing was stopped with an intracoronary injection of 50–200 *μ*g of nitroglycerin (Millisrol®; G. Pohl-Boskamp, Hohenlockstedt, Germany).

### 2.5. Isolation of Monocytes from Human Peripheral Blood Mononuclear Cells

Following overnight fasting just before coronary angiography, blood was collected in BD Vacutainer® CPT™ mononuclear cell preparation tubes (#362753, BD Diagnostics, Sparks Glencoe, MD, USA) and centrifuged at 1800 × *g* at room temperature for 20 min. After removing the upper layer containing plasma and Ficoll™Hypaque™ and without disturbance of the red lowest layer, the opaque interface containing the mononuclear cells was carefully transferred to a new 50 mL conical tube. The mononuclear cells were washed twice with phosphate-buffered saline (PBS). Subsequently, monocytes were isolated using Invitrogen™ Dynabeads® CD14 superparamagnetic beads (#11149D, Thermo Fisher Scientific Inc., Waltham, MA, USA) and magnetic activated cell sorting (MACS). Isolated monocyte purity was assessed by flow cytometry of fluorescein-labeled CD14-positive cells. Finally, isolated monocytes were resuspended in Invitrogen™ TRIzol™ reagent (#15596026, Thermo Fisher Scientific Inc., Waltham, MA, USA), and the total RNA extract was stored at −80°C until use.

### 2.6. Differentiation of Monocytes to Macrophages

For differentiation of monocytes to macrophages, monocytes were enriched by allowing adherence in 5% CO_2_ atmosphere incubator at 37°C for 2 h. While nonadherent cells with the supernatant were carefully discarded, adherent monocytes were carefully washed with prewarmed 15 mL PBS and washing solution aspirated. Thereafter, the ImmunoCult™-SF macrophage medium (#10961, STEMCELL Technologies Inc., Kent, WA, USA) was used for monocyte differentiation to macrophages following manufacturer's instruction. M1 macrophages were obtained by treatment with 10 ng/mL lipopolysaccharides (LPS) (#L2630, Sigma-Aldrich Corporation, St. Louis, MO, USA) and 5 U/mL human recombinant interferon- (IFN-) *γ* (#I17001, Sigma-Aldrich Corporation, St. Louis, MO, USA), while M2 macrophages were obtained by treatment with 20 ng/mL human recombinant IL-4 (#I4269, Sigma-Aldrich Corporation, St. Louis, MO, USA). Then, the M1 or M2 cells were incubated in 5% CO_2_ atmosphere incubator at 37°C for 24 h, supernatant discarded, and fresh RPMI-1640 (Sigma-Aldrich Corporation, St. Louis, MO, USA) supplemented with 5% FBS, 2 mM glutamine, 50 *μ*g/mL streptomycin, 50 U/mL penicillin, and 0.05 mM *β*-mercaptoethanol (*β*-ME) for expansion.

### 2.7. Lp(a) Isolation and Detection

Lp(a) was isolated from pooled plasma sample from healthy subjects (*n* = 7) with Lp(a) > 50 mg/dL. The isolation of Lp(a) from the pooled plasma was carried out strictly as previously described [48]. The concentration of Lp(a) was measured using the Human Lipoprotein an ELISA Kit (ab212165, Abcam plc., Cambridge, UK); the lower limit of detection was 17.2 ng/mL.

### 2.8. RNA Isolation and Quantitative Real-Time Polymerase Chain Reaction (qRT-PCR)

RNA extraction and qRT-PCR were performed as previously described [8]. The specific primer sequences used are as follows: human *α*7-nAChR (forward: 5′-GGC AGA TAT CAG TGG CTA TAT C-3′, reverse: 5′-CTT CAT TCG CAG GAA CC-3′); human IL-6 (forward: 5′-CCA GCT ATG AAC TCC TTC TC-3′, reverse: 5′-GCT TGT TCC TCA CAT CTC TC-3′); and human GAPDH (forward: 5′-ACC CAC TCC TCC ACC TTT GA-3′, reverse: 5′-CTG TTG CTG TAG CCA AAT TCG T-3′). The PCR product amplification procedure is as follows:1 cycle at 95°C for 10 min, followed by 40 cycles at 95°C for 30 s, 57°C for 1 min, and 72°C for 1 min. Postamplification melting curve analysis was performed to verify amplicon accuracy. GAPDH served as the internal control.

### 2.9. Proinflammatory IL-6 Cytokine Assay and Fluorescence-Activated Cell Sorting (FACS) Flow Cytometry

After 15 min fixation of 2 × 10^6^ cells in 4% formaldehyde (pH 7.5) at room temperature, the cells were incubated in blocking solution containing 1% bovine serum albumin (#A7030, Sigma-Aldrich Corporation, St. Louis, MO, USA) and 1% goat serum in PBS for 30 min, followed by 2 h incubation in primary antibodies against *α*7-nAChR (1 : 100, Abcam) or Lp(a) (1 : 100, #ab125014, Abcam plc.). After washing twice with PBS, cells were incubated in PBS/fluorescein isothiocyanate-conjugated IgG solution for 1 h and then cell surface marker expression levels analyzed using the BD FACSCalibur™ modular analyzer (BD Biosciences, San Jose, CA, USA). The concentration of intracellular IL-6 was measured using the sandwich Human Interleukin-6 DuoSet ELISA Development Immunoassay Kit (#DY206, R&D Systems Inc., Minneapolis, Minnesota); the lower limit of detection was 0.70 pg/mL.

### 2.10. NO Analytical Measurements

The NO level was detected using ready kits (Abcam, Co., Cambridge, MA, USA; ab65328) following the manufacturer's protocols. Briefly, the nitrate is catalyzed with nitrate reductase into nitrite. Later, total nitrite is converted into a deep purple azo compound (azo chromophore) with Griess Reagents. The absorbance of the purple azo compound is measured at 540 nm, where the absorbance of the azo compound is directly proportional to NO production. The detection limit of the assay is approximately 1 nmol nitrite/well or 10 *μ*M.

### 2.11. *α*7-nAChR Luciferase Activity Reporter Assay

Both monocyte-derived macrophages were stably transfected with *α*7-nAChR luciferase reporter plasmids (GeneCopoeia Inc., Rockville, MD, USA) containing an Invitrogen™ pcDNA™3.1^(+)^-derived neomycin-resistant thymidine kinase (TK) cassette (pCHRNA7neo-luc) (#V79020, Thermo Fisher Scientific Inc., Waltham, MA, USA). The viable transfected (resistant) cells were expanded and subcultured severally (12 passages) in neomycin containing RPMI-1640. For the *α*7-nAChR luciferase activity reporter assay, after pretreating cells with 500 nM LDL or Lp(a) for 30 min, treatment media were decanted, cells were washed with ice-cold 1x PBS thrice, lysed with passive lysis 5x buffer from the luciferase assay system (#E1941, Promega Corporation, Fitchburg, WI, USA), and then, the cell lysates were used to determine the *α*7-nAChR luciferase activity following the manufacturer's protocol.

### 2.12. Western Blot Assays

Western blot analyses were performed according to standard protocol [49] using the following antibodies against: *α*7-nAChR (ab216485; 1 : 1000), p38 (ab31828; 1 : 1000), p-p38 (phospho T180+Y182) (ab4822; 1 : 1000), IL-6 (ab9324; 1 : 1000), inducible NO synthase (ab3523; 1 : 1000), and GAPDH (ab9484; 1 : 10,000), purchased from Abcam (Abcam plc., Cambridge, UK), and RhoA (#2117; 1 : 1000), RhoA-GTP (#8820; 1 : 1000), ROCK1 (#4035; 1 : 1000), ROCK2 (#9029; 1 : 1000), t-MBS (#2634; 1 : 1000), and p-MBS (#3040; 1 : 1000) from Cell Signaling Technology (Cell Signaling, Danvers, MA, USA) in Supplementary Table S1. The protein bands were pictured using enhanced chemiluminescence detection system (Amersham Pharmacia Biotech, NJ, USA) and quantified using ImageJ software (https://imagej.nih.gov/ij/).

### 2.13. Immunofluorescence

Briefly, 5 × 10^3^ cells were plated into 6-well plates containing 1–2 mL medium. After 24–36 h, Lp(a) was added and cells were incubated for another 48 h. For nicotinic acetylcholine receptor staining, cells were incubated with either Alexa Fluor 488 *α*-bungarotoxin, a competitive antagonist to nAChR (*α*-BTX, green fluorescence; B-13422, Thermo Fisher Scientific Inc., Waltham, MA, USA), or *α*7-nAChR antibody (red fluorescence; 21379-1-AP, Thermo Fisher Scientific Inc., Waltham, MA, USA). For the staining of nuclei, sections and/or cells were incubated with 50 *μ*g/mL DAPI in PBS and then mounted with an antifade mounting medium (0.1 M Tris, pH 9.0).

### 2.14. Short Hairpin RNA (shRNA) Transfection

The shRNA specifically targeting CHRNA7 was using the Nicotinic Acetylcholine Receptor Alpha 7 Human shRNA Plasmid Kit (Locus ID 1139) (ORIGENE, Rockville, MD, USA). For CHRNA7 silencing, HCASMCs grown to ~80% confluence were transfected with CHRNA7 shRNA. Lipofectamine 3000 (Invitrogen, Carlsbad, CA, USA) was used for the transfection of the shRNAs according to the manufacturer's protocol. The total RNA or protein was extracted 48 h after transfection and used for the western blot analyses.

### 2.15. Statistical Analysis

All assays were performed at least 3 times in triplicates. Continuous variables are expressed as the mean ± standard deviation (SD) or median (2-quartile), and positively skewed variables were log-transformed for subsequent intergroup Student's *t*-test. Discrete variables were expressed as numbers and percentages (%) of the total sample and comparisons made using the two-tailed Fisher's exact test, while categorical variables were analyzed using the *χ*^2^ test. Correlation between the levels of serum Lp(a) and hs-CRP, IL-6, or *α*7-nAChR expression in patients with CAS were determined by Spearman correlation. The coefficient of determination (*r*^2^) and associated *p* value were calculated using linear regression analysis. All statistical analyses were performed with SPSS (IBM Corp., released 2017, IBM SPSS Statistics for Windows, Version 25.0, Armonk, NY: IBM Corp.). A two-tailed *p* value < 0.05 was considered statistically significant.

## 3. Results

### 3.1. Study Cohort Baseline Characteristics

A total of 64 patients were enrolled in the study (median age = 58.0 years; interquartile range, 49.2–65.0 years). Patients in the CAS group compared with patients in the control group had significantly higher Lp(a) levels (*p* = 0.011) (Table 1). The leukocyte, monocyte, and macrophage count and hs-CRP values were also significantly higher in the CAS group than in the control group. Single-vessel spasm was the most common finding in the CAS patients, and spasm was provoked mostly in the right coronary artery. No difference in medication use before coronary angiography was observed between the 2 groups. However, after coronary angiography, the number of patients being treated with calcium channel blockers and nitrates was significantly higher in the CAS group than in the control group (Table 1).

### 3.2. Lp(a) Levels Positively Correlate with Monocytic *α*7-nAChR Levels and Are Implicated in Inflammation-Associated CAS

**Figure 1:**
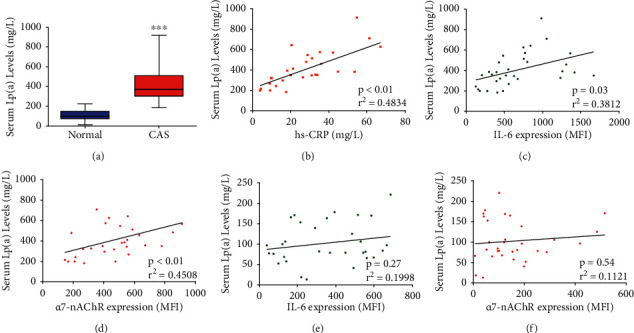
Lp(a) levels positively correlate with patient monocytic *α*7-nAChR levels and are implicated in inflammation-associated CAS. (a) Box and whisker plot of the differential serum Lp(a) levels in control subjects and patients with CAS. Spearman dots and regression line plots showing the correlation between serum Lp(a) levels and (b) hs-CRP levels, (c) IL-6 expression, or (d) monocytic *α*7-nAChR expression, in patients with CAS. Spearman dots and regression line plots showing the correlation between serum Lp(a) levels and (e) IL-6 expression or (f) monocytic *α*7-nAChR expression, in the controls. *R*^2^: coefficient of determination; MFI: median fluorescence intensity; ^∗^*p* < 0.05, ^∗∗^*p* < 0.01, and ^∗∗∗^*p* < 0.001.

Lp(a) levels were significantly higher in patients with CAS than in the control group (*p* = 0.011) (Table 1, Figure 1(a)). There was a significantly positive correlation between Lp(a) and hs-CRP (*r*^2^ = 0.48, *p* < 0.01) (Figure 1(b)) or IL-6 (*r*^2^ = 0.38, *p* = 0.03) (Figure 1(c)). Furthermore, Lp(a) levels were positively correlated with *α*7-nAChR expression (*r*^2^ = 0.45, *p* < 0.01) in patients with CAS (Figure 1(d)). However, the correlation between Lp(a) and IL-6 (*r*^2^ = 0.20, *p* = 0.27) or *α*7-nAChR (*r*^2^ = 0.11, *p* = 0.54) (Figures 1(e) and 1(f)) was markedly reduced in the control group.

### 3.3. The Apolipoprotein (a) Component of Lp(a) Interacts with and Induces *α*7-nAChR Expression in the Monocyte-Derived Macrophages of Patients with CAS

**Figure 2:**
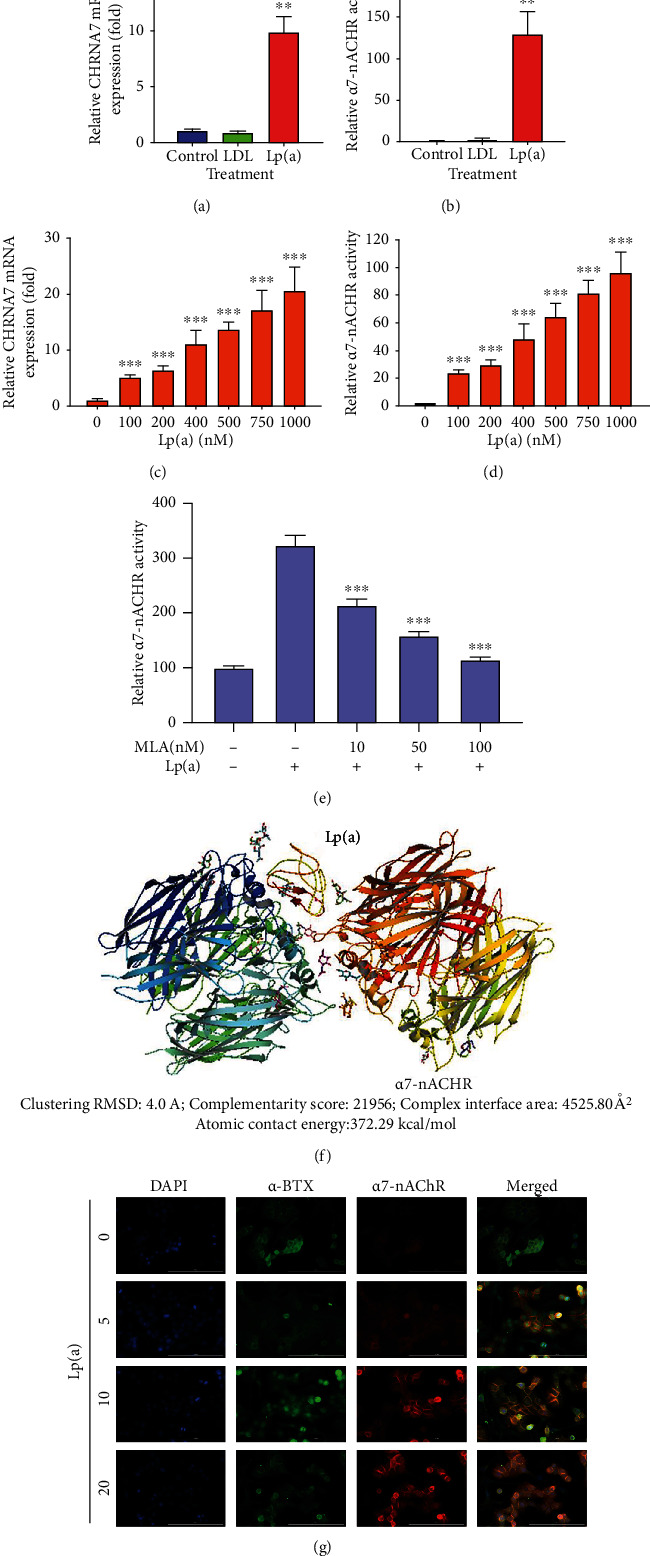
The apolipoprotein (a) component of Lp(a) interacts with and induces *α*7-nAChR expression in the monocyte-derived macrophages of patients with CAS. Graphical representation of the effect of 500 nM LDL or Lp(a) on the (a) relative expression of CHRNA7 mRNA or (b) relative luciferase reporter activity of *α*7-nAChR in the patient monocyte-derived macrophages. Histograms showing the effect of 100 nM–1000 nM Lp(a) on the (c) relative expression of CHRNA7 mRNA or (d) relative luciferase reporter activity of *α*7-nAChR in the patient monocyte-derived macrophages. (e) Methyllycaconitine (MLA) dose dependently inhibited the Lp(a)-induced activation of *α*7-nAChR. (f) Visualization of the direct molecular interaction between Lp(a) and *α*7-nAChR using the PyMoL molecular docking and visualization software. Complex formation criteria are indicated. (g) Immunofluorescence demonstrated induced expression of *α*7-nAChR after Lp(a) treatment and fluorescent-protein tagging showed a high correlation for protein localization with *α*-BTX. Original magnification ×200. DAPI (blue) served as a nuclear marker. ^∗^*p* < 0.05, ^∗∗^*p* < 0.01, and ^∗∗∗^*p* < 0.001. Lp(a): lipoprotein(a); CHRNA7: gene encoding *α*7-nAChR.

Compared with the untreated control or the treatment of 500 nM LDL, exposure to 500 nM Lp(a) induced ~10-fold increase (*p* < 0.01) in the expression levels of *α*7-nAChR mRNA in CAS patient monocyte-derived macrophages (Figure 2(a)). Lp(a) significantly induced higher *α*7-nAChR activity in the CAS monocyte-derived macrophages than in the LDL-treated or untreated cells (~126-fold, *p* < 0.01) (Figure 2(b)). In parallel assays, exposure to 100 nM–1 *μ*M Lp(a) increased dose-dependently (*p* < 0.001) *α*7-nAChR mRNA expression level (Figure 2(c)) and luciferase activity (Figure 2(d)) in CAS monocyte-derived macrophages. Methyllycaconitine (MLA) dose dependently inhibited the Lp(a)-induced activation of *α*7-nAChR (Figure 2(e)). By using the Edu PyMoL molecular graphics system version 1.7.4, based on a clustering root mean squared deviation of 4.0 A, we demonstrated that the ligand-binding domain of pentameric *α*7-nAChR directly interacts with the kringle KIV_7_, KIV_10_, and KV domains of the apolipoprotein (a) component of Lp(a) with a geometric shape complementarity score of 21,956, a complex interface area of ~4525.80 Å^2^, and an atomic contact energy of 372.29 kcal/mol (Figure 2(f)). Immunofluorescence image demonstrated Lp(a)-induced expression of *α*7-nAChR, which had a high correlation with *α*7-nAChR protein localization with *α*-BTX using fluorescent-protein tagging, original magnification ×200. DAPI (blue) served as a nuclear marker (Figure 2(g)).

### 3.4. Lp(a) Preferentially Induces Patient Monocyte-Derived Macrophage M1 Polarization

**Figure 3:**
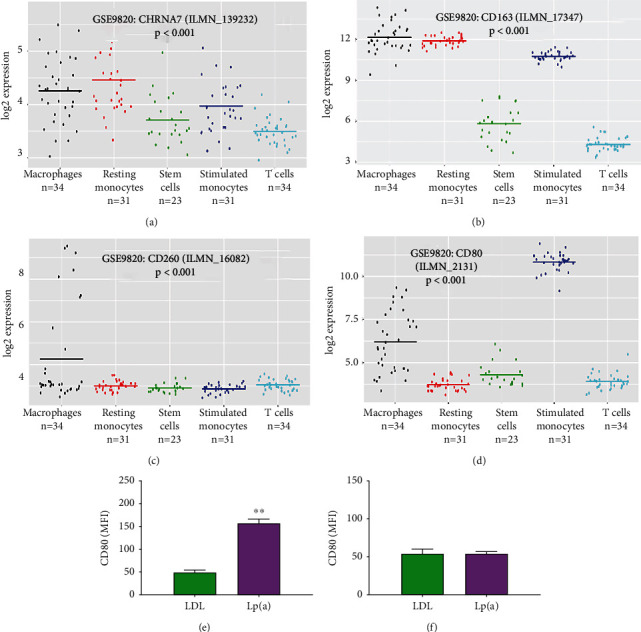
Lp(a) preferentially induces patient monocyte-derived macrophage M1 polarization. Dots and line plot showing the expression profile of (a) CHRNA7, (b) CD163, (c) CD206, or (d) CD80 in the macrophages, resting monocytes, stem cells, stimulated monocytes, and T cells of patients with CAD using the GSE9820/GPL6255/GDS3690 dataset, *n* = 153. Flow cytometry cell count polygons (*upper panel*) and histograms (*lower panel*) depicting the effect of treatment with 500 nM LDL or Lp(a) on the (e) CD80 median fluorescence intensity or (f) CD206 median fluorescence intensity of CAS monocyte-derived macrophages. Histogram colors are green for control antibody and purple for target antibody. The macrophages were exposed to either Lp(a) or LDL for 24 hours. LDL: low-density lipoprotein; PE: phycoerythrin. ^∗^*p* < 0.05, ^∗∗^*p* < 0.01, and ^∗∗∗^*p* < 0.001.

To evaluate if and how Lp(a) modulates macrophage activities in CAS, which has not been studied previously, we used CAD-specific functional genomics data from the National Center for Biotechnology Information Gene Expression Omnibus website (https://www.ncbi.nlm.nih.gov/geo/) to perform comparison of knowledge between CAD and CAS. Our reanalysis of the GSE9820/GPL6255/GDS3690 dataset (*n* = 153), which originally analyzed various circulating mononuclear cells from patients with severe CAD, revealed that the expression of *α*7-nAChR, CD163, CD206, and CD80 was the highest in macrophages, compared to the CD14+ resting monocytes, CD34+ stem cells, LPS-stimulated monocytes, or CD4+ T helper cells (Figures 3(a) and 3(b)). In addition, the median CD80+ macrophage population was 1.49-fold more than the CD206+ macrophages (Figures 3(c) and 3(d)). In our CAS monocyte-derived macrophages, 500 nM Lp(a) compared with the 500 nM LDL elicited a 3.16-fold stronger shift in fluorescence intensity of the CD80+ macrophage population (*p* < 0.01) (Figure 3(e)). However, exposure to neither LDL nor Lp(a) had any apparent effect on the median fluorescence intensity (MFI) of the CD206+ macrophage population (Figure 3(f)), suggesting that Lp(a) preferentially induces M1 macrophage polarization in patients with CAS.

### 3.5. Lp(a) Promotes Inflammation in Patient Monocyte-Derived Macrophages and HCASMCs by Inducing *α*7-nAChR-Dependent Activation of p38 MAPK Signaling

**Figure 4:**
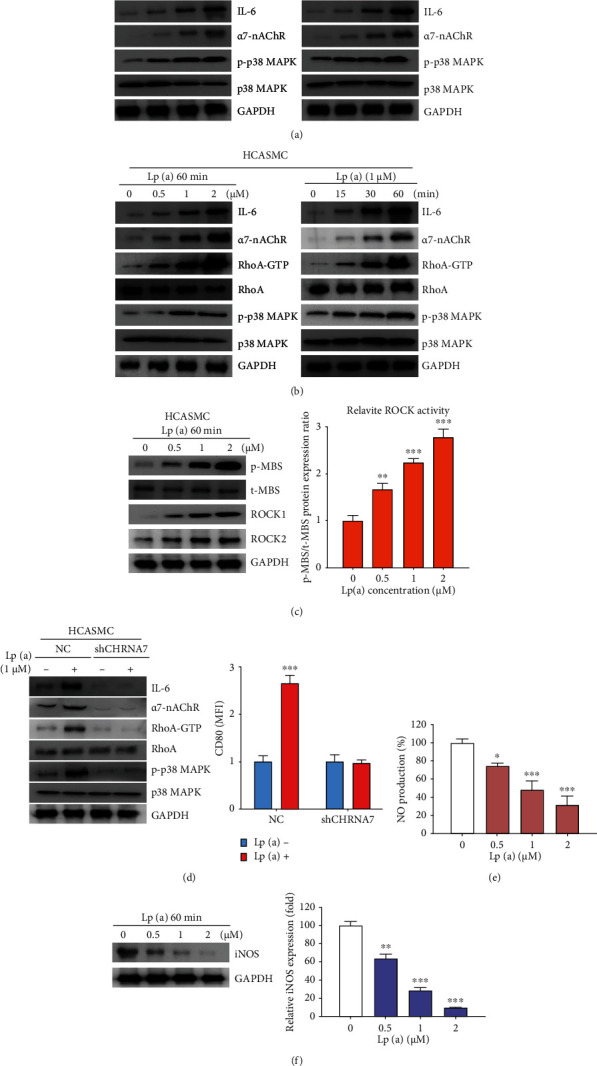
Lp(a) promotes inflammation in PMDMs and HCASMCs by inducing *α*7-nAChR-dependent activation of p38 MAPK signaling. (a) Representative western blot photo images showing the effect of treating patient monocyte-derived macrophages with 0.5 *μ*M–2 *μ*M Lp(a) for 60 min (*upper panel*) or with 1 *μ*M Lp(a) at 15, 30, and 60 min time points (*lower panel*), on *α*7-nAChR, IL-6, p-p38 MAPK, and p38 MAPK protein expression levels. (b) Representative western blot photo images showing the effect of treating HCASMCs with 0.5 *μ*M–2 *μ*M Lp(a) for 60 min (*upper panel*) or with 1 *μ*M Lp(a) at 15, 30, and 60 min time points (*lower panel*), on RhoA-GTP, RhoA, p-p38 MAPK, and p38 MAPK protein expression levels. (c) Representative western blot photo images showing that treating HCASMCs with 0.5 *μ*M–2 *μ*M Lp(a) for 60 min increases ROCK activity dose dependently. (d) Representative western blot images showing how shCHRNA7 affects the expression of RhoA-GTP, RhoA, *α*7-nAChR, p-p38 MAPK, and p38 MAPK in HCASMCs in the presence or absence of 1 *μ*M Lp(a). Histograms show the effect of shCHRNA7 on CD80 MFI in HCASMCs in the presence or absence of 1 *μ*M Lp(a). (e) PMDMs were treated with different concentrations of Lp(a) (0-2 *μ*M) and the nitric oxide production was measured. (f) Lp(a) treatment dose dependently reduced the iNOS expression level in PMDMs. HCASMC: human coronary artery smooth muscle cell; MFI: median fluorescence intensity; PMDM: patient monocyte-derived macrophage; RhoA: Ras-homologous A; ROCK: Rho-kinase; shCHRNA7: *α*7-nAChR-targeting short hairpin RNA. ^∗^*p* < 0.05, ^∗∗^*p* < 0.01, and ^∗∗∗^*p* < 0.001; GAPDH served as loading control.

Exposure to Lp(a) significantly increased the macrophage expression of *α*7-nAChR, phosphorylated p38 (p-p38) MAPK, and IL-6 proteins in a dose-dependent and time-dependent manner (Figure 4(a)). A similar dose- and time-dependent induction of the expression of *α*7-nAChR/p38 MAPK/IL-6/RhoA-GTP was observed in HCASMCs (Figure 4(b)). To further understand the influence of Lp(a) on the Rho GTPase, we examined the activation of an important Rho GTPase, RhoA-GTP, and its downstream effector, ROCK. Lp(a) dose dependently activated ROCK (Figure 4(c)). While treatment with 1 *μ*M Lp(a) enhanced the expression of *α*7-nAChR, p-p38 MAPK, and RhoA-GTP protein in unsilenced negative control HCASMCs by ~4-fold, ~3.2-fold, and ~2.5-fold, respectively, compared to the untreated cells, its enhancing effect on *α*7-nAChR, p-p38 MAPK, and RhoA-GTP protein level was significantly reduced by shRNA of CHRNA7 function (shCHRNA7) (Figure 4(d)), suggesting that the activation of p38 MAPK signaling in macrophages and HCASMCs by Lp(a) is *α*7-nAChR-dependent. On the other hand, while shCHRNA7 exhibited no suppressive effect on the CD80+ M1 macrophages in the absence of Lp(a), exposure to Lp(a) significantly enhanced the ability of shCHRNA7 to suppress the fluorescence intensity of CD80+ M1 cells (1.73-fold, *p* < 0.01) (Figure 4(d)). When patient monocyte-derived macrophages were treated with increasing concentrations of Lp(a) (0 to 2 *μ*M), the NO production and the expression of inducible NO synthase were dose-dependently inhibited (Figures 4(e) and 4(f)).

### 3.6. The Human Monoclonal Antibody, Tocilizumab, Disrupts Lp(a)-Induced *α*7-nAChR/p38 MAPK Signaling by Attenuating Inflammation in Patient Monocyte-Derived Macrophages and HCASMCs

**Figure 5:**
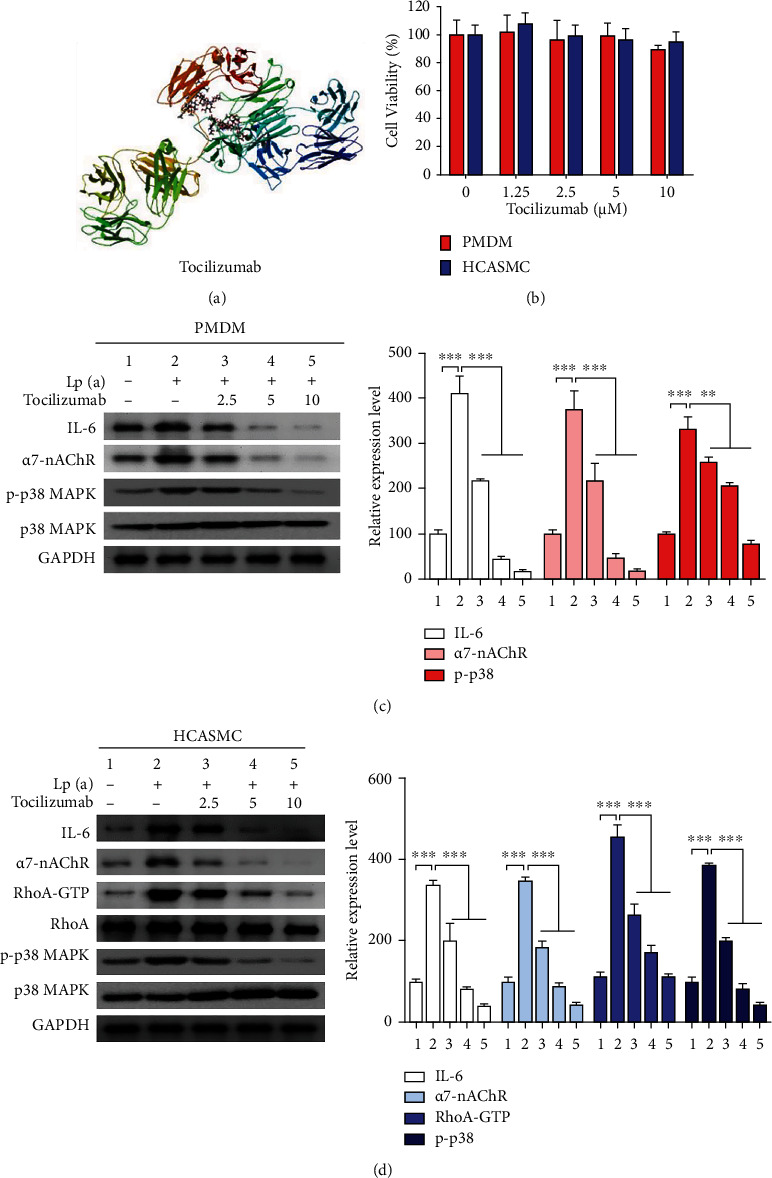
The human monoclonal antibody, tocilizumab, disrupts Lp(a)-induced *α*7-nAChR/p38 MAPK signaling by attenuating inflammation in patient monocyte-derived macrophages and HCASMCs. (a) 3D chemical structures of tocilizumab with molecular formula C_6428_H_9976_N_1720_O_2018_S_42_ and molar mass 144987.06 g/mol. (b) Graphical representation of the effect of 1.25 *μ*M–10 *μ*M tocilizumab on the viability of HCASMCs or PMDMs. Representative western blot photo images and histograms showing how treatment with 1 *μ*M Lp(a) and/or 2.5 *μ*M–10 *μ*M tocilizumab affects the expression of *α*7-nAChR, p-p38, and p38 proteins in (c) patient monocyte-derived macrophages or in (d) HCASMCs. ^∗^*p* < 0.05, ^∗∗^*p* < 0.01, and ^∗∗∗^*p* < 0.001; GAPDH served as loading control. PMDM: patient monocyte-derived macrophage.

For the rational selection of therapeutic monoclonal antibodies to examine whether Lp(a) could be a potential target, tocilizumab, a 148 g/mol anti-IL-6 receptor monoclonal antibody with the chemical structure C_6428_H_9976_N_1720_O_2018_S_42_, was used (Figure 5(a)). Treatment with 1.25 *μ*M–10 *μ*M tocilizumab exhibited no observable toxic effects to the patient monocyte-derived macrophages and HCASMCs (Figure 5(b)). Furthermore, exposure to 2.5 *μ*M–10 *μ*M tocilizumab dose dependently and significantly reversed the Lp(a)-induced upregulation of *α*7-nAChR, p-p38 MAPK protein expression levels in both patient monocyte-derived macrophages and HCASMCs, and additionally RhoA-GTP in HCASMCs (Figures 5(c) and 5(d)).

## 4. Discussion

In this translational work, we demonstrated that elevated serum Lp(a) levels were positively correlated with the levels of CRP, IL-6, and monocytic *α*7-nAChR in CAS. Lp(a), through its apolipoprotein (a) chain, increased the expression of *α*7-nAChR in the monocyte-derived macrophages of patients with CAS and HCASMCs. Lp(a), in synergism with *α*7-nAChR, induced the proinflammatory activation of patient monocyte-derived M1 macrophages and HCASMCs through p38 MAPK/IL-6/RhoA-GTP. Lp(a) dose dependently reduced the inducible NO synthase expression level in monocyte-derived macrophages derived from CAS patients. Tocilizumab, a monoclonal antibody against IL-6 receptor, reduced Lp(a)-associated expression of *α*7nAChR-dependent activation of p38 MAPK, IL-6, and additionally RhoA-GTP in HCASMCs. To the best of our knowledge, this is the first study demonstrating the mechanism by which interactions of Lp(a)/monocyte/HCASMC and the subsequent expression of *α*7-nAChR/p38 MAPK/IL-6/RhoA-GTP contribute to VSMC dysfunction and the development of CAS.

The diagnosis of CAS by coronary angiography in the catheterization laboratory was not rare in the 1970s and 1980s. It soon became clear that CAS could occur in patients with atherosclerotic obstructive CAD [50, 51], nonobstructive CAD, or angiographically normal coronary arteries [3]. Hence, coronary lesions are dynamic [52]. Among the mechanisms of angina pectoris, CAS had long been considered the chief one [53], although it was yet unproved until 1940s when the observation of angina-associated fixed atherosclerotic stenosis at autopsy led to a revision of the theory that CAS may produce paroxysmal myocardial ischemia [54–56]. Lp(a) is now established as an independent risk factor for myocardial infarction and ischemic heart disease [57]. Genetic studies have provided strong evidence of causality; however, the disease-causing mechanism is to some extent still unknown [57]. Besides, considering CRP from a genetic perspective, investigators have found that specific polymorphisms in the CRP gene associate with plasma levels of CRP and predict future events, suggesting a potentially causal link between CRP and atherothrombosis [58]. While Lp(a) appears to be a largely inherited basis for premature atherogenesis, a very different process to that of CAS, the relationship between Lp(a) levels and inflammation, as reflected by elevated CRP levels, is somewhat unclear [57]. Previous studies have only investigated this association in highly selected groups such as patients with rheumatoid arthritis [59, 60] or patients undergoing hemodialysis [61]. Notably, Lp(a) levels from the Danish general population are minimally increased at increased levels of CRP [57]. Regarding the differential development of atherosclerosis and CAS, smoking and CRP have been demonstrated to be the 2 important risk factors for both diseases [3, 62, 63]. Therefore, it remains unknown whether they represent a risk continuum of atherosclerosis or completely different diseases. On the other hand, there is no clear boundary between stable and unstable angina and some overlap must be taken into account in the natural history of CAD [64]. As a result, dynamic stenosis can be caused by (1) “physiologic” increase of coronary tone, as in stable angina, (2) spasm, as in variant angina, and (3) thrombosis, usually combined with “physiologic” changes in tone or with spasm, or both, as in unstable angina [1]. Hence, this “atherosclerotic continuum” has been proposed as one of the most promising research target [65]. Furthermore, studies of genetic mutations or polymorphisms in the pathogenesis of CAS have been inconsistent [66]. Mutations or polymorphisms of the endothelial NO synthase gene [10, 67, 68] and polymorphisms of paraoxonase I gene [69] are significantly associated with CAS. However, NO gene polymorphisms are found in only one-third of patients [70]. Gene polymorphisms of other proteins that have been described in CAS contain adrenergic and serotoninergic receptors [71, 72], angiotensin-converting enzyme [73], and inflammatory cytokines [74, 75]. In a Japanese cohort analysis, the NADH/NADPH oxidase p22 phox gene is a predisposition locus in men, while stromelysin-1 and interleukin-6 genes are predisposition loci in women [76]. Although CAS itself is rarely familial and family history is not a risk factor for CAS, there is familial evidence of CAS and possible involvement of HLA-DR2 linkage disequilibrium with a susceptibility gene of CAS in a Japanese study [77]. Familial migraine and CAS in 2 siblings have also been reported [78]. As researchers at or associated with the National Human Genome Research Institute unlock the secrets of the human genome (the complete set of human genes), nearly all diseases have a genetic component [79]. In addition, the fluctuations of CAS activity appear with circadian variations in the short term and active and inactive phases in the long term [80], suggesting gene-environment interactions may exist in the development of CAS [76].

Although Lp(a) levels are largely determined by genetic factors, Lp(a) is also induced by mediators of the innate immunity in several chronic inflammatory diseases such as rheumatoid arthritis [81] and Crohn's disease [82] and in patients undergoing hemodialysis [61], which could be responsible for the increased cardiovascular risk found in such subjects [82]. Moreover, there is evidence that Lp(a) levels also increase with other conditions such as surgery or myocardial infarction [83], all possibly being associated with induction of the innate immunity. While in subjects with CAD there is a lack of correlation of Lp(a) with CRP [84], Lp(a) levels from the Danish general population are minimally increased at increased levels of CRP [57], which has been demonstrated to be an important risk factor for CAS. Because the correlation of Lp(a) with CRP is significant in our CAS patients, it suggests a different mechanism from CAD that leads to CAS. Lp(a) levels have been demonstrated to be more correlated to IL-6 compared with metabolic parameters, such as body mass index, insulin resistance, and triglyceride, indicating that Lp(a) serum concentrations are not only genetically determined but are also influenced by IL-6 [85]. Notwithstanding that the correlation of Lp(a) with *α*7-nAChR in humans has not been evaluated and requires further exploration, a positive feedback may exist between the 2 markers.

Lp(a) exerts both proatherogenic and prothrombotic effects, parts of which are primarily related to the LDL component whereas others are apolipoprotein (a)-dependent [86]. The competition with plasminogen for binding to endothelial cells and monocytes is mediated by apolipoprotein (a) [87], which supports a procoagulant/antifibrinolytic function for apolipoprotein (a), but there has been little progress in proving the pathophysiological relevance of the binding in humans. In transgenic mouse aorta, elevated plasma level of apolipoprotein (a) or Lp(a) alone does not cause endothelial dysfunction [88], suggesting that either the plasma levels were too low or, more importantly, other factors are needed to observe the phenomenon, which seems to be supported by studies in adult humans [88] and children with familial hypercholesterolemia [89], where impaired endothelium-dependent vasodilatation was observed in the presence of multiple risk factors in addition to elevated plasma levels of Lp(a). Indeed, Lp(a) only exists in monkeys, apes, and humans [86]. While some species lack KV, human apolipoprotein (a) kringles are specialized domains to mediate ligand interactions [90], often with lysine-containing substrates, as it contains both KV and an intact lysine binding site in KIV_10_. Therefore, human Lp(a) is exceptionally pathogenic. Even though a cognate Lp(a) receptor has not been identified, several other receptors interact with L(a) either via its apolipoprotein B, apolipoprotein (a), or oxidized phospholipid components [91]. The roles of these receptors, including lipoprotein receptors, toll-like and scavenger receptors, lectins, and plasminogen receptors, remain unclear [91]. While the uptake of oxidized low-density lipoprotein in macrophages is mediated through *α*7-nAChR [92], a similar interaction of Lp(a) with *α*7-nAChR in CAS patient monocyte-derived macrophages is observed. Obviously, mechanistic studies are required to determine the role of apolipoprotein (a)/Lp(a) in the interaction with *α*7-nAChR and the molecular basis for resultant increased risk in CAS development.

The provocative testing nowadays involves the use of ergonovine or acetylcholine [70]. In many countries, including Taiwan and the United States, only ergonovine is available for the diagnosis of CAS. Ergonovine, which is used to control postpartum uterine bleeding, was discovered in 1949 to provoke angina and was proposed in 1963 as a diagnostic test for coronary disease [93]. In normal coronary arteries, only mild widespread vasoconstriction (<20% diameter reduction) would be induced [94]. Ergonovine testing in the catheterization laboratory was used in the late1970s and early 1980s to identify the mechanism of chest pain when nonobstructive coronary artery disease was found by angiography. CAS is diagnosed when any one of the following conditions is present such as (1) spontaneous attacks, (2) positive non-drug-induced CAS provocation test (e.g., hyperventilation test and exercise test), or (3) positive drug-induced CAS provocation test (e.g., acetylcholine and ergonovine provocation test) [95]. While the frequency of provoked CAS by the intracoronary administration is about 2.5-fold higher than that by the intravenous administration of ergonovine and acetylcholine [96, 97], there is no difference regarding the incidence of provoked CAS between ergonovine and acetylcholine [98]. Provoked CAS by ergonovine tends to be proximal and focal, whereas CAS provoked by acetylcholine is distal and diffuse [99–101]. Although the intracoronary injection of ergonovine and acetylcholine provoked CAS in 65% and 80% in a previous study [100], respectively, no differences existed regarding the provoked CAS between intracoronary ergonovine and acetylcholine in a later study [101]. The efficacy of intracoronary administration of acetylcholine in doses of 10 to 100 *μ*g is comparable to methylergonovine [70, 102, 103]. Of note, besides invasive diagnosis of CAS, ergonovine echocardiography has been used in Korea for noninvasive diagnosis of CAS [104]. Further studies are needed to evaluate the differences of coronary response between the ergonovine and acetylcholine examinations.

Acetylcholine, ergonovine, serotonin, and histamine cause endothelium-dependent vasodilation by stimulating NO release from the normal endothelium, and they can induce CAS in the presence of endothelial dysfunction [105]. Dysfunctional endothelial NO synthase and therefore deficient release of NO have been shown to be significantly associated with CAS [10, 106]. Furthermore, NO deficiency has been shown in the nonspastic coronary arteries as well as in the peripheral arteries, indicating that NO deficiency may occur in the entire vascular system in patients with CAS [107]. Remarkably, while neither stimulated NO synthesis nor basal NO production and release in endothelium seems to be impaired by elevated Lp(a) concentrations, the endothelium-dependent vasoconstrictive response to N-monomethyl L-arginine is enhanced in patients with high Lp(a) plasma levels [108]. Although oxidized Lp(a), but not native Lp(a), inhibits inducible NO synthesis in lipopolysaccharide/interferon stimulated mouse macrophages in a dose-dependent manner [109], we demonstrated for the first time that the inducible NO synthesis and the subsequent NO production in our CAS patient monocyte-derived macrophages were dose-dependently inhibited by Lp(a), suggesting a role of inducible NO synthase in CAS development and the effects on inducible NO synthesis by Lp(a) may be cell type selective. While NO is produced by different cell types and important in regulating smooth muscle relaxation [110], the activation of inducible NO synthase varies depending on cell types and species [111]. Furthermore, NO plays critical roles in immune suppression [112]. Inducible NO synthase-deficient mice than wild-type mice are more susceptible to the development of inflammatory diseases such as experimental allergic encephalitis [113]. Although endothelial NO synthase is the only NOS expressed in normal vascular endothelium, during inflammation, blood vessels express both inducible and endothelial NO synthase [114]. Moreover, inducible NO synthase produced in rabbit carotid arteries counteracts VSMC contraction by activation of soluble guanylate cyclase [115]. In pigs, NO produced by inducible NO synthase in the coronary VSMCs exerts an inhibitory and vasculoprotective effect against the cytokine-induced proliferative/vasospastic changes of the coronary artery in vivo [116]. In addition, inducible NO synthase is a signature molecule for M1 macrophages [117]. Thus, a complete understanding of the molecular mechanisms involved in the regulation of M1 innate immune responses should provide insights into the pathogenesis and treatment of CAS.

To differentiate Kounis syndrome from nonallergic CAS, the understanding of individual hypersensitivity is vitally important. While a relation exists between white blood cell counts and the incidence of coronary heart disease in epidemiologic studies [118], elevated peripheral white blood cell and monocyte counts, hs-CRP, interleukin-6, and adhesion molecules have been demonstrated in CAS patients [119, 120]. Although the eosinophil counts predict the severity of CAS, CAS can also result in an increase in eosinophil counts during follow-up in patients with CAS [118]. In our previous nationwide population-based cohort study showing the important role of CAS, regardless of sex, as a risk factor for incident diabetes, peripheral monocyte and eosinophil counts were borderline insignificant and significantly higher in nondiabetic CAS patients than nondiabetic non-CAS patients, respectively, in a single hospital substudy [121]. In addition, aspirin-induced eosinophilia-associated coronary artery vasospasm (EACAV) is a generalized terminology associated with various eosinophilic disorders such as aspirin-exacerbated respiratory disease that varies in presentation [122]. Of most EACAV patients, all were middle-aged, refractory to traditional antianginal therapy, and responsive to oral steroids [122]. While the allergic inflammatory response starts when an allergen activates the tissue resident mast cell, triggering the release of various granule-stored and newly formed mediators, as the inflammation progresses, a chronic allergic inflammation always features prominent tissue eosinophilia [123]. The interactions due to such “allergic effector unit” may modulate the severity and/or duration of the allergic inflammatory reaction [123]. Taken together, while monocytes play an important role in CAS, eosinophils and mast cells appear to be more important than monocytes in mediating nondiabetic CAS and allergic CAS, respectively. Future studies have to better delineate which patients benefit most from a measurement of differential cell counts to assess the development of CAS.

While the expression data related to macrophage polarization have previously highlighted interspecies discrepancy [124], few data are available regarding human monocyte-to-macrophage differentiation and polarization to M1 and M2 upon exposure to Lp(a). Although in atherosclerotic lesions, cytokines can modify macrophage phenotypes, such as M1 and M2 [125], in disease contexts, M1 macrophages are implicated in initiating and sustaining inflammation [126] and can therefore be detrimental to health. Lp(a) elicits the proinflammatory response in healthy monocytes in vitro, an effect markedly attenuated by inactivating oxidized phospholipids present on apolipoprotein (a) [127]. Furthermore, in CAD, elevated Lp(a) levels compared with normal Lp(a) levels increase the expression levels of the scavenger receptors CD36 on monocytes, which is correlating to Lp(a) levels, whereas the expression of other receptors such as CD163 and CD206 was not different [127]. A similar phenomenon was observed that Lp(a) increases the expression of phenotypical M1 marker CD80 via *α*7-nAChR in our CAS monocyte-derived macrophages, indicating that 2 different vascular pathologies may exist in CAS and CAD. On the other hand, in human monocytes and monocyte-derived dendritic cells, the upregulation of *α*7-nAChRs and M1 marker CD40/CD86 enhances adaptive immunity in atherosclerosis, including T cell proliferation and cytokine production [128]. The similar upregulation of *α*7-nAChRs and M1 marker CD80 in our CAS patient monocyte-derived macrophages is of functional relevance for eicosanoid production and may contribute to pathophysiological reactions in CAS. Moreover, a hallmark of M1 polarization is the synthesis of IL-6 [129]. Similar to oxidized LDL [130], but not native LDL, Lp(a) diminished apoptosis of the activated macrophages. Hence, the upregulation of *α*7-nAChRs and M1 marker is important in both adaptive and innate immunity. In response to inflammatory stimuli, an afferent signal via the vagus nerve is triggered, activating efferent responses to attenuate tissue-specific cytokine production by the activation of the *α*7-nAChR in macrophages. Notably, spontaneous episodes of CAS in patients are often preceded by a decrease of vagal activity [131]. In addition, many studies using murine atherosclerotic models have either described an anti- or proatherogenic role of the *α*7-nAChR, which is still an area of debate in the literature [41]. The role of *α*7-nAChR in distinct immune cells may differ depending on cell type and function. In macrophages, besides decreasing the release of inflammatory cytokines, *α*7-nAChR stimulates the survival and polarization of the anti-inflammatory M2 phenotype [132], supporting the notion that immune cells have their own cholinergic system. However, we observed that Lp(a) polarized macrophages toward the M1 phenotype and subsequently increased IL-6 production. Because M1 macrophages are involved in inflammatory responses by producing chemokine ligands and proinflammatory cytokines, such as tumor necrosis factor-*α* and IL-6 for immune stimulation [133], our findings suggest that Lp(a)/M1 macrophage/IL-6 pathway contributes to the development of CAS. Hence, Lp(a) may modulate the acetylcholine-related cellular environment in an autocrine/paracrine way via *α*7-nAChR expressed by macrophages. Furthermore, activation of VSMC *α*7-nAChR has been reported to increase IL-6 following prior nicotine exposure [134]. This finding is insightful not only because it links increased IL-6 expression with *α*7-nAChR activation but also because it suggests the role of *α*7-nAChRs in vascular immunogenicity. Based on our findings, it seems reasonable to suggest that the interaction between Lp(a) and *α*7-nAChR significantly increases IL-6 levels, which ultimately prove critical during acute coronary syndrome. Although IL-6 has been implicated in the pathogenesis of atherosclerosis [135], in vitro studies have demonstrated that the activation of *α*7-nAChRs attenuates the release of IL-6 by macrophages [136], and the level of IL-6 is increased in patients with CAS [119]. Because different doses of nicotine can lead to activation or desensitization of nAChR function [137], complex interactions may exist between *α*7-nAChRs and smoking in atherosclerosis, while the role of these interactions in CAS development is currently unknown. Therefore, Lp(a) exposure *α*7-nAChR activation may increase IL-6 levels through undetermined mechanisms, which requires further exploration. Collectively, these observations suggest the different effects of Lp(a) and *α*7-nAChR on IL-6 production in CAS from their effects on atherosclerotic CAD. These properties of cells of the monocyte–macrophage lineage may represent a target for therapeutic exploitation.

The pathophysiological role of Lp(a) in humans is still not fully elucidated. While plasma concentrations of Lp(a) are observed to rise acutely under pathological challenge, for example, after myocardial infarction and percutaneous coronary intervention [138], it has been demonstrated that prolonged exposure to high-circulating apolipoprotein (a) levels would render the VSMCs more stationary and contractile [138]. Taken together, it appears that Lp(a), acting as an acute phase reactant, induces the activation of RhoA-GTP and ROCK, potentially leading to the development of CAS. Our finding that the downstream effector pathway by which Lp(a) activated monocyte-derived macrophages and HCASMC relied on the *α*7nAChR-dependent activation of p38 MAPK is consistent with the effect of *α*7-nAChR in dendritic cells [128]. Furthermore, Lp(a) has been demonstrated to activate endothelial cells through activation of intracellular p38 MAPK signaling pathway [138]. In human apolipoprotein (a) transgenic rabbits, the atherosclerotic lesions are predominantly enriched in VSMCs, suggesting that Lp(a) promotes the proliferation of immature or activated VSMCs [139]. Notably, we found that *α*7-nAChR was involved in the activation of the RhoA-GTP and downstream effectors ROCKs in HCASMCs, which facilitates VSMC dysfunction [17]. Consistent with our prior study [15], increased levels of ROCK activity in HCASMCs are associated with CAS. Furthermore, in a cellular study, using a small interfering RNA approach, selective suppression of ROCK2 expression significantly attenuated VSMC contraction by modulating myosin phosphatase activity [140], suggesting increased expression of ROCK2 could lead to CAS. Our findings complement and extend these previous studies that Lp(a) signals through *α*7-nAChR/p38 MAPK to activate CAS patient monocyte-derived macrophages and HCASMCs. Further studies are needed to clarify the role of these relationships in mediating the development of CAS.

Although aspirin at low doses decreases Lp(a) levels slightly, there are currently no pharmaceutical treatments, including lipid-lowering strategies, available for the reduction of the effects of Lp(a) and hence a greater understanding of the mechanisms underlying its functional effects on monocyte-macrophage and VSMC may provide alternative therapeutic targets. Recently, although the inflammatory hypothesis of cardiovascular disease was demonstrated in 2 large-scale multicenter randomized clinical trials using either a selective IL-1*β* antagonist [141] or low-dose colchicine [142], its clinical application using other affordable mainstream anti-inflammatory therapies remains challenging [143]. Emerging clinical and translational data suggest a synergism between the effects of Lp(a) and systemic inflammation [127]. Tocilizumab, a monoclonal antibody against IL-6 receptor approved for the treatment of rheumatoid arthritis in 2009 in Europe [90], lowers Lp(a) serum levels in rheumatoid arthritis patients by up to 50% [144]. The up- and downregulation of *α*7-nAChR expression on immune T cells has been found to be under the influence of interleukin cytokines in inflammatory bowel disease, which is nicotine-mediated and smoking-related [145]. In our study, tocilizumab reduced Lp(a)-associated expression of *α*7nAChR and the receptor-dependent activation of p38 MAPK, IL-6, and additionally RhoA-GTP in HCASMCs, suggesting a direct and specific pathogenic effect of Lp(a). Collectively, these observations implicate that interleukin cytokines themselves can alter the function of *α*7-nAChR. RhoA-GTP and its downstream effector, Rho-kinase/ROCK, inhibit myosin light chain phosphatase, leading to augmentation of *myosin light chain* phosphorylation and the subsequent VSMC contraction in response to vasoconstrictor stimuli. Hence, one may consider anticytokine IL-6 as a new promising treatment of elevated Lp(a) levels in affected patients. However, despite these recent advances, it needs further studies to examine which patient populations would benefit the most from Lp(a) reduction and what degree of Lp(a) lowering would be required to demonstrate incremental clinical benefit despite the use of established medical therapies [146, 147].

In our study, all 64 patients, who had chest pain and suspected ischemic heart disease on noninvasive tests and no angiographic evidence of obstructive CAD, were subjected to intracoronary methylergonovine testing. In the Asymptomatic Cardiac Ischemia Pilot (ACIP) study, asymptomatic patients with CAS or CAD were those without symptoms to indicate myocardial ischemia [148]. While screening asymptomatic patients for the presence of CAS or CAD may potentially impact therapeutic management and outcome, the approach to asymptomatic patients with suspected CAS or CAD is based on the history and/or electrocardiographic (ECG) evidence of myocardial ischemia or an abnormal noninvasive test [149]. It is recognized that when tested, a subgroup of these asymptomatic patients will have transient abnormalities consistent with myocardial ischemia, which is termed silent ischemia, and the abnormalities detected may consist of reversible ECG ST-segment shifts on exercise testing or ambulatory monitoring, perfusion abnormalities on radionuclide scans (i.e., stress 201Tl, sestamibi, and PET) or regional wall motion abnormalities during left ventricular imaging (i.e., stress echocardiography or radionuclide ventriculography). Thus, the absence of symptoms does not necessarily mean the absence of either ischemia or an adverse prognosis. Diabetes, old age, females, hypertension, polyneuropathy, and cardiac transplantation, when accompanied by significant CAD, are all associated with a high frequency of myocardial ischemia without symptoms [70]. However, multiple guidelines and scientific statements have discouraged the use of ambulatory monitoring, treadmill testing, stress echocardiography, stress myocardial perfusion imaging, and electron-beam computed tomography as routine screening tests in asymptomatic individuals [149]. Furthermore, because a diagnosis of CAS cannot be directly established based on symptoms [97], standard 12-lead electrocardiography results [150], ambulatory monitoring of electrocardiography [27], or exercise testing [151], and invasive coronary angiography with provocative testing are the gold standard method of diagnosing CAS [70], direct referral for diagnostic coronary angiography may be indicated in symptomatic patients with chest pain possibly attributable to myocardial ischemia when noninvasive testing is contraindicated or unlikely to be adequate due to illness, disability, or physical characteristics [152]. The diagnosis of angina associated with diabetes can be particularly difficult because of the paucity of symptoms of myocardial ischemia due to autonomic and sensory neuropathy, and a lowered threshold for coronary angiography is appropriate [152]. Therefore, with only a few exceptions, coronary angiography is not indicated in asymptomatic patients with suspected CAS or CAD, unless noninvasive testing reveals findings that suggest a high risk for adverse outcome [70]. While our study was not a randomized controlled trial and the inclusion of a group of gender- and age-matched asymptomatic individuals referred for diagnostic coronary angiography was not approved by the Taipei Medical University Joint Institutional Review Board, future studies including gender- and age-matched asymptomatic individuals will help elucidate the role of Lp(a) in defining the severity and susceptibility of inflammation-associated CAS. Further investigation is required to better delineate these relationships. In our study, CAS patients were typically middle-aged men, often smokers, which is typical in East Asia, especially in Japan [153]. However, there are not enough data on the prevalence of CAS both in the Eastern and Western countries, probably because it is difficult to examine CAS systematically at each time of coronary angiography [27]. CAS appears to be more common in Caucasian than Japanese women [154], and there is lower incidence of smoking among the whole of CAS females than among males [155]. Hence, among Caucasians, CAS is not rare among youngish women, usually nonsmokers [156]. Recently, the presence of CAS is more frequent in Caucasians when invasive coronary angiography with provocative testing is aggressively performed [157]. Thus, the aggressive effort of making a diagnosis of CAS may help clarify the real prevalence of CAS worldwide.

CAS is a multifactorial disease involving the contribution of both vascular wall- and blood-related factors in pathogenesis. Precipitating factors may trigger the onset of CAS and cause angina in the same patient under different conditions [153]. Moreover, VSMC hyperreactivity can cause CAS through various pathways [6]. Considering endothelial cell-smooth muscle cell coculture systems, they are sufficiently developed such that they are mainly employed for high-throughput screening applications in atherosclerotic vascular wall remodeling [158]. Several different approaches of the coculture systems are available to identify drugs and targets for angiogenesis [158]. Although direct contact coculture systems provide several distinct advantages, they still need more development so that a normal intima can be produced, and the cells can be exposed to both stretch and fluid flow [158]. Microplate and microfluidic systems can be utilized to produce high-throughput identification formats of lead candidates [158]. Vascular endothelium responds specifically to arterial fluid shear stress but less so to pressure or cyclic stretch [159]. Steady or pulsatile laminar shear stresses cause the endothelium to align in the direction of flow, release vasodilators, reduce their growth rate, increase their elastic modulus, and increase expression of anti-inflammatory genes. In contrast, low and oscillating shear stresses promote the release of vasoconstrictors and the expression of proinflammatory and oxidative stress genes [159, 160]. Laminar shear stress applied to endothelial cells exert atheroprotective functions by modulating the underlying VSMCs from synthetic to contractile phenotype [161]; however, contractile rather than synthetic phenotype VSMCs play a main role in the pathogenesis of CAS [6]. Furthermore, laminar shear stress has anti-inflammatory effects by inhibiting VSMC-induced proinflammatory responses in endothelial cells. To date, no information is available concerning the effects of Lp(a) on monocyte-derived macrophages in patients with CAS and HCASMCs. We, therefore, analyzed the protein expression levels of Lp(a) and *α*7-nAChR in the monocytes of patients with CAS. Furthermore, we investigated the effects of Lp(a) on monocyte-to-macrophage differentiation and polarization based on CD80 or CD206 positivity and *α*7-nAChR-dependent activation of the p38 MAPK signaling in monocyte-derived macrophages and primary HCASMCs. In addition, we previously demonstrated the negative effects of diabetes mellitus and hypertension on CAS development in patients with high CRP levels, indicating 2 different vascular pathologies exist in CAS and atherosclerotic cardiovascular disease [6]. Collectively, more development is needed before applying endothelial cell-smooth muscle cell coculture systems to research in CAS. Nonetheless, endothelial cell-smooth muscle cell coculture systems should be utilized in the future studies of aberrant endothelial cell-smooth muscle cell communication in CAS. In our ongoing studies we aim to identify the nature of endothelial cell–VSMC crosstalk, which may provide the key cellular and molecular mechanisms of CAS-related vascular wall remodeling.

In the study of a *cause* of *disease*, Koch's postulates were invaluable when they were developed and remain largely valid for a few defined circumstances. Koch's postulates were initially developed in the 19th century to establish microorganism function and were modified in the 20th century to include methods to establish molecular causality [162]. Although isolation of the pathogen from the diseased host is the gold standard of the postulates, rigorously applying Koch's postulates to the etiology of CAS has several limitations. First, smoking that may not induce CAS in some people with low CRP levels can become a risk factor and potentially pathogenic for CAS in other people with high CRP levels [163]. Second, there are experimental animal models evaluating the causal role of Lp(a) in atherosclerosis and aortic stenosis, but not in CAS. Finally, something that may be useful in proving causality is whether eradication of the pathogen results in cure, which is not described in Koch's postulates. Although Lp(a) is not expressed in commonly studied laboratory animals, mouse and rabbit models transgenic for Lp(a) and apo(a) have been developed to study their pathogenicity in vivo, which have provided significant insights into the pathophysiology of Lp(a) in mediating atherosclerosis [164]. While apo(a) is retained in atheromas in mouse models and suggests that it promotes fatty streak formation and Lp(a) promotes atherosclerosis and vascular calcification in rabbit models, many of these models have limitations [164]. Because apo(a) is not covalently linked to mouse apoB to form Lp(a), mouse models need to be transgenic for both apo(a) and human apolipoprotein B-100 [164]. In established mouse and rabbit atherosclerotic models, Lp(a) levels are low, usually <20 mg/dL, which is within the normal range in humans [164]. Furthermore, only one apo(a) isoform can be expressed in a given model whereas more than 40 isoforms exist in humans [164]. It is ideal for mouse models to be studied for atherosclerosis in an LDL receptor negative background, as mice do not develop sufficiently elevated plasma cholesterol to form atherosclerosis [164]. As such, the development of optimized Lp(a) transgenic animal models will advance the understanding of the mechanistic role of Lp(a) in atherosclerosis and aortic stenosis [164], as well as in CAS, and provide a platform to examine novel therapies for cardiovascular disease. On the other hand, controversy exists regarding whether CRP is only a clinically useful determinant of disease or whether it also may play a causal role in the atherothrombotic process [165, 166]. Although much information has been provided by past studies, CRP cardiovascular biology remains largely observational, with few studies showing cause and effect relationships, which note that CRP induces endothelial cell activation and dysfunction, has substantive effects on VSMCs and neointimal formation, and directly affects monocyte and macrophage activity as well as matrix metalloproteinase function [167]. Furthermore, human CRP infusion studies show both proinflammatory and prothrombotic effects [168], whereas in transgenic mouse models, CRP seems to increase thrombosis rates only after vascular injury [169]. In a recent prospective study, Ridker and colleagues report on a monoclonal antibody targeting anti-interleukin 1*β* (anti-IL-1*β*) on cardiovascular events in humans [141]. The authors argue that this study [141] fulfills Koch's postulates for ASCVD since inflammation, including the proinflammatory cytokine, IL-1*β*, has been shown in animal models to contribute to atherosclerosis, and now, this study shows that blocking IL-1 with the 150 mg dose in humans results in a significant decrease in cardiovascular events [170]. Collectively, the “marker versus mechanism” debate remains open and is an area with a need for more research, including a need to develop novel Lp(a) and CRP inhibitors that can be used to test directly whether Lp(a) and CRP reduction results in reduced event rates [167]. Debate concerning mechanistic properties of Lp(a) and CRP to fulfill the Koch's postulates before being useful in a clinical setting should have little bearing on their utilities as clinically effective biomarkers for risk detection [167]. In our study, we provided a framework of investigating Lp(a) in association with CAS to ensure that scientific rigor is applied when proposing a mechanistic role of Lp(a) in the development of CAS.

Our study has some limitations. Firstly, the relatively small cohort size (*n* = 64) might be difficult to establish causality. Secondly, the use of certain medications, including beta blockers [171], statins [172], Ca^2+^-channel blockers [173], or nitrates [174], which are known to affect IL-6 or *α*7-nAChR expression and/or activity to varying extents, is a putative limitation. Thirdly, the presence of confounders that could have affected the accurate measurement of patients' cytokine, Lp(a), or *α*7-nAChR level is probable. Finally, translating findings to subjects without cigarette exposure history but who used other nicotine-containing products, e.g., Swedish SNUS, might have constituted some limitation.

## 5. Conclusion

**Figure 6:**
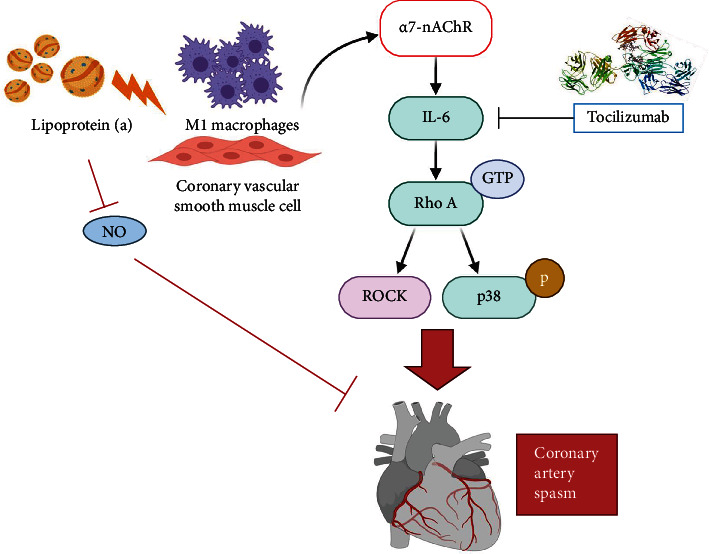
Graphical abstract depicting how Lp(a)-triggered inflammation drives CAS through macrophage M1 polarization, activation of coronary VSMC, and *α*7-nAChR/p38 MAPK signal induction. Tocilizumab disrupts Lp(a)-induced *α*7-nAChR/p38 signaling by attenuating the inflammation in coronary VSMCs and patient monocyte-derived macrophages.

Serum Lp(a) levels are positively correlated with the levels of CRP, IL-6, and monocytic *α*7-nAChR in CAS. Lp(a) induces macrophage M1 polarization and, through its apolipoprotein (a) chain, the expression of *α*7-nAChR/p38 MAPK/IL-6 and dose dependently inhibited the NO production and the expression of inducible NO synthase in the monocyte-derived macrophages of patients with CAS. Lp(a) activates HCASMCs via *α*7-nAChR/p38 MAPK/IL-6/RhoA-GTP signal induction. Tocilizumab reduces the interaction of Lp(a)/monocyte/HCASMC and the subsequent expression of *α*7-nAChR/p38 MAPK/IL-6/RhoA-GTP (Figure 6), suggesting *α*7-nAChR partly under the influence of IL-6 and anti-cytokine IL-6 as a promising treatment of CAS. Our study provides a new avenue in understanding the process of *α*7-nAChR-induced VSMC dysfunction and shows promise in the development of potential therapeutic agents for CAS.

## Figures and Tables

**(a):** 

	Controls (*n* = 32)	CAS (*n* = 32)	*p* value
Age (years)	62.5 (54.0–66.5)	53.5 (46.3–63.8)	0.07
Male sex, *n* (%)	24 (75)	21 (66)	0.41
Body mass index (kg/m^2^)	27.3 ± 4.2	27.2 ± 3.4	0.86
Current smoker, *n* (%)	9 (28)	10 (31)	0.78
Diabetes mellitus, *n* (%)	9 (28)	3 (9)	0.06
Hypertension, *n* (%)	17 (53)	11 (34)	0.13
Left ventricular ejection fraction, %	65 ± 6	66 ± 6	0.74
Total cholesterol (mg/dL)	173 ± 33	191 ± 40	0.05
Triglyceride (mg/dL)	131 ± 100	170 ± 219	0.37
LDL cholesterol (mg/dL)	107 ± 26	119 ± 42	0.15
HDL cholesterol (mg/dL)	44 ± 16	43 ± 9	0.83
Lipoprotein(a) (mg/dL)	102 ± 49	406 ± 164	0.011
Peripheral leukocytes (/mm^3^)	6066 ± 1671	6994 ± 1943	0.045
Monocytes (/mm^3^)	465 ± 183	568 ± 220	0.046
Macrophage (/mm^3^)	120 ± 16	397 ± 147	0.013
Lymphocytes (/mm^3^)	1673 ± 615	2036 ± 886	0.06
Hemoglobin (g/dL)	13.9 ± 1.4	14.0 ± 1.8	0.85
Hematocrit (%)	41.3 ± 6.4	40.4 ± 5.2	0.54
Platelet (×10^3^/mm^3^)	217 ± 57	242 ± 62	0.1
hs-CRP (mg/L^∗^)	0.80 (0.50–2.28)	1.04 (1.00–2.03)	0.044
Coronary artery with lesion			
Left anterior descending artery, *n* (%)		4 (11)	
Left circumflex artery, *n* (%)		3 (9)	
Right coronary artery, *n* (%)		25 (78)	
Number of spastic arteries			
One-vessel spasm, *n* (%)		27 (84)	
Two-vessel spasm, *n* (%)		4 (14)	
Three-vessel spasm, *n* (%)		1 (4)	

**(b):** 

Medications	A	D	A	D	A	D
*β*-Blockers, *n* (%)	21 (66)	13 (41)	21 (66)	6 (19)	1.0	0.06
Calcium-channel blockers, *n* (%)	16 (50)	18 (56)	11 (34)	31 (97)	0.21	<0.001
Angiotensin receptor blocker, *n* (%)	17 (53)	19 (59)	11 (34)	12 (38)	0.13	0.08
Nitrates, *n* (%)	16 (50)	5 (16)	10 (31)	13 (41)	0.13	0.03
Statins, *n* (%)	6 (19)	13 (41)	11 (34)	20 (63)	0.16	0.08
Aspirin, *n* (%)	30 (94)	26 (81)	30 (94)	31 (97)	1.0	0.05
Diuretics, *n* (%)	2 (6)	2 (6)	0 (0)	0 (0)	0.15	0.15

Values are expressed as *mean* ± *SD* or median (interquartile range). A: before angiography; ^∗^CAS: coronary artery spasm; D: at discharge; hs-CRP: high-sensitivity C-reactive protein; LDL: low-density lipoprotein; HDL: high-density lipoprotein; Lp(a): lipoprotein(a). ^∗^Log-transformed values were used for the analyses.

## Data Availability

The datasets used and analyzed in the current study are publicly accessible as indicated in the manuscript.

## Additional Points

*Highlights*. (1) Apolipoprotein (a)/lipoprotein(a) induces inflammatory *α*7-nAChR/p38 MAPK/IL-6/RhoA-GTP signaling axis in coronary artery spasm. (2) Apolipoprotein (a)/lipoprotein(a)-triggered inflammation modulates coronary artery spasm through M1 macrophage polarization. (3) The *α*7-nAChR on macrophages could represent a promising new therapeutic target in coronary artery spasm.

## Supplementary Materials

Supplementary 1 Supplementary Table S1: western blot antibody sheet.

Supplementary 2 Supplementary Figure S1: full-size blots of Figures 2(a) and 2(b).

Supplementary 3 Supplementary Figure S2: full-size blots of Figures 2(c), 2(d), and 2(f).

Supplementary 4 Supplementary Figure S3: full-size blots of Figures 4(c) and 4(d).

## References

[1] Maseri A. (1987). Role of coronary artery spasm in symptomatic and silent myocardial ischemia. *Journal of the American College of Cardiology*.

[2] Antonio L. G., Giulia C., Filippo C. (2011). Mechanisms of coronary artery spasm. *Circulation*.

[3] Hung M.-Y., Kounis N. G., Lu M.-Y., Hu P. (2020). Myocardial ischemic syndromes, heart failure syndromes, electrocardiographic abnormalities, arrhythmic syndromes and angiographic diagnosis of coronary artery spasm: literature review. *International Journal of Medical Sciences*.

[4] Hung M.-J., Kuo L.-T., Cheng C.-W., Chang C.-P., Cherng W.-J. (2004). Comparison of peripheral monocyte counts in patients with and without coronary spasm and without fixed coronary narrowing. *The American Journal of Cardiology*.

[5] Park J. Y., Rha S.-W., Li Y.-J. (2013). The impact of high sensitivity C-reactive protein level on coronary artery spasm as assessed by intracoronary acetylcholine provocation test. *Yonsei Medical Journal*.

[6] Hung M.-J., Hsu K.-H., Hu W.-S., Chang N.-C., Hung M.-Y. (2013). C-reactive protein for predicting prognosis and its gender-specific associations with diabetes mellitus and hypertension in the development of coronary artery spasm. *PLoS One*.

[7] Hung M.-J., Cherng W.-J., Cheng C.-W., Li L.-F. (2006). Comparison of serum levels of inflammatory markers in patients with coronary vasospasm without significant fixed coronary artery disease versus patients with stable angina pectoris and acute coronary syndromes with significant fixed coronary artery disease. *The American Journal of Cardiology*.

[8] Fichtlscherer S., Breuer S., Schächinger V., Dimmeler S., Zeiher A. M. (2004). C-reactive protein levels determine systemic nitric oxide bioavailability in patients with coronary artery disease. *European Heart Journal*.

[9] Hung M.-Y., Wu Y.-H., Bamodu O. A. (2018). Activation of the monocytic *α*7 nicotinic acetylcholine receptor modulates oxidative stress and inflammation-associated development of coronary artery spasm via a p38 MAP-kinase signaling-dependent pathway. *Free Radical Biology and Medicine*.

[10] Kugiyama K., Yasue H., Okumura K. (1996). Nitric oxide activity is deficient in spasm arteries of patients with coronary spastic angina. *Circulation*.

[11] Roy A., Guatimosim S., Prado V. F., Gros R., Prado M. A. M. (2015). Cholinergic activity as a new target in diseases of the heart. *Molecular Medicine*.

[12] Santanam N., Thornhill B. A., Lau J. K. (2012). Nicotinic acetylcholine receptor signaling in atherogenesis. *Atherosclerosis*.

[13] Hoover D. B. (2017). Cholinergic modulation of the immune system presents new approaches for treating inflammation. *Pharmacology & Therapeutics*.

[14] Duris K., Lipkova J., Jurajda M. (2017). Cholinergic anti-inflammatory pathway and stroke. *Current Drug Delivery*.

[15] Hung M.-J., Cherng W.-J., Hung M.-Y. (2012). Increased leukocyte Rho-associated coiled-coil containing protein kinase activity predicts the presence and severity of coronary vasospastic angina. *Atherosclerosis*.

[16] Strassheim D., Gerasimovskaya E., Irwin D., Dempsey E. C., Stenmark K., Karoor V. (2019). RhoGTPase in vascular disease. *Cells*.

[17] Liang D., Wang Z., Yan Z. (2017). Nicotine facilitates VSMC dysfunction through a miR-200b/RhoGDIA/cytoskeleton module. *Scientific Reports*.

[18] Bertrand D., Lee C.-H. L., Flood D., Marger F., Donnelly-Roberts D. (2015). Therapeutic potential of *α*7 nicotinic acetylcholine receptors. *Pharmacological Reviews*.

[19] Miwa K., Nakagawa K., Yoshida N., Taguchi Y., Inoue H. (2000). Lipoprotein(a) is a risk factor for occurrence of acute myocardial infarction in patients with coronary vasospasm. *Journal of the American College of Cardiology*.

[20] Nishino M., Mori N., Yoshimura T. (2014). Higher serum uric acid and lipoprotein(a) are correlated with coronary spasm. *Heart Vessels*.

[21] Albers J. J., Slee A., O’Brien K. D. (2013). Relationship of apolipoproteins A-1 and B, and lipoprotein(a) to cardiovascular outcomes: the AIM-HIGH trial (Atherothrombosis Intervention in Metabolic Syndrome with Low HDL/High Triglyceride and Impact on Global Health Outcomes). *Journal of the American College of Cardiology*.

[22] Galle J., Stunz P., Schollmeyer P., Wanner C. (1995). Oxidized LDL and lipoprotein(a) stimulate renin release of juxtaglomerular cells. *Kidney International*.

[23] Rothe G., Stöhr J., Fehringer P., Gasche C., Schmitz G. (1997). Altered mononuclear phagocyte differentiation associated with genetic defects of the lysosomal acid lipase. *Atherosclerosis*.

[24] Si M. L., Lee T. J. (2002). Alpha7-nicotinic acetylcholine receptors on cerebral perivascular sympathetic nerves mediate choline-induced nitrergic neurogenic vasodilation. *Circulation Research*.

[25] Picciotto M. R., Kenny P. J. (2013). Molecular mechanisms underlying behaviors related to nicotine addiction. *Cold Spring Harbor Perspectives in Medicine*.

[26] Ottervanger J. P., Festen J. M., de Vries A. G., Stricker B. H. (1995). Acute myocardial infarction while using the nicotine patch. *Chest*.

[27] Hung M. Y., Hsu K. H., Hu W. S., Chang N. C., Huang C. Y., Hung M. J. (2013). Gender-specific prognosis and risk impact of C-reactive protein, hemoglobin and platelet in the development of coronary spasm. *International Journal of Medical Sciences*.

[28] Patel K. V. (2008). Variability and heritability of hemoglobin concentration: an opportunity to improve understanding of anemia in older adults. *Haematologica*.

[29] Segal J. B., Moliterno A. R. (2006). Platelet counts differ by sex, ethnicity, and age in the United States. *Annals of Epidemiology*.

[30] Robertson R. M., Robertson D., Friesinger G. C., Timmons S., Hawiger J. (1980). Platelet aggregates in peripheral and coronary-sinus blood in patients with spontaneous coronary-artery spasm. *The Lancet*.

[31] Folts J. D., Stamler J., Loscalzo J. (1991). Intravenous nitroglycerin infusion inhibits cyclic blood flow responses caused by periodic platelet thrombus formation in stenosed canine coronary arteries. *Circulation*.

[32] Loscalzo J. (1985). N-Acetylcysteine potentiates inhibition of platelet aggregation by nitroglycerin. *Journal of Clinical Investigation*.

[33] Imam H., Nguyen T. H., Stafford I. (2021). Impairment of platelet NO signalling in coronary artery spasm: role of hydrogen sulphide. *British Journal of Pharmacology*.

[34] Forman M. B., Oates J. A., Robertson D., Robertson R. M., Roberts L. J., Virmani R. (1985). Increased adventitial mast cells in a patient with coronary spasm. *The New England Journal of Medicine*.

[35] Kounis N. G. (2013). Coronary hypersensitivity disorder: the Kounis syndrome. *Clinical Therapeutics*.

[36] Vieira-Alves I., Coimbra-Campos L. M. C., Sancho M., da Silva R. F., Cortes S. F., Lemos V. S. (2020). Role of the *α*7 nicotinic acetylcholine receptor in the pathophysiology of atherosclerosis. *Frontiers in Physiology*.

[37] Paré G., Çaku A., McQueen M. (2019). Lipoprotein(a) Levels and the risk of myocardial infarction among 7 ethnic groups. *Circulation*.

[38] Chang N. C., Yeh C. T., Lin Y. K. (2021). Garcinol attenuates lipoprotein(a)-induced oxidative stress and inflammatory cytokine production in ventricular cardiomyocyte through *α*7-nicotinic acetylcholine receptor-mediated inhibition of the p38 MAPK and NF-*κ*B signaling pathways. *Antioxidants*.

[39] Kanwar S. S., Stone G. W., Singh M. (2016). Acute coronary syndromes without coronary plaque rupture. *Nature Reviews Cardiology*.

[40] Scalone G., Niccoli G., Crea F. (2019). Editor’s choice-pathophysiology, diagnosis and management of MINOCA: an update. *European Heart Journal. Acute Cardiovascular Care*.

[41] Miwa K., Ishii K., Makita T., Okuda N. (2004). Diagnosis of multivessel coronary vasospasm by detecting postischemic regional left ventricular delayed relaxation on echocardiography using color kinesis. *Circulation Journal*.

[42] Kvietys P. R., Granger D. N. (2012). Role of reactive oxygen and nitrogen species in the vascular responses to inflammation. *Free Radical Biology and Medicine*.

[43] Li C., Ma Q., Toan S., Wang J., Zhou H., Liang J. (2020). SERCA overexpression reduces reperfusion-mediated cardiac microvascular damage through inhibition of the calcium/MCU/mPTP/necroptosis signaling pathways. *Redox Biology*.

[44] Herman A. B., Silva Afonso M., Kelemen S. E. (2019). Regulation of stress granule formation by inflammation, vascular injury, and atherosclerosis. *Arteriosclerosis, Thrombosis, and Vascular Biology*.

[45] Grainger D. J., Kirschenlohr H. L., Metcalfe J. C., Weissberg P. L., Wade D. P., Lawn R. M. (1993). Proliferation of human smooth muscle cells promoted by lipoprotein(a). *Science*.

[46] Gibbons R. J., Chatterjee K., Daley J. (1999). ACC/AHA/ACP-ASIM guidelines for the management of patients with chronic stable angina: executive summary and recommendations. A Report of the American College of Cardiology/American Heart Association Task Force on Practice Guidelines (Committee on Management of Patients with Chronic Stable Angina). *Circulation*.

[47] JCS Joint Working Group (2010). Guidelines for diagnosis and treatment of patients with vasospastic angina (coronary spastic angina) (JCS 2008): digest version. *Circulation Journal*.

[48] Sharma M., Von Zychlinski-Kleffmann A., Porteous C. M., Jones G. T., Williams M. J. A., McCormick S. P. A. (2015). Lipoprotein (a) upregulates ABCA1 in liver cells via scavenger receptor-B1 through its oxidized phospholipids. *Journal of Lipid Research*.

[49] Liu Z.-Q., Mahmood T., Yang P.-C. (2014). Western blot: technique, theory and trouble shooting. *North American Journal of Medicine and Science*.

[50] Prinzmetal M. (1959). A variant form of angina pectoris; preliminary report. *The American Journal of Medicine*.

[51] Mac Alpin R. N., Kattus A. A., Alvaro A. B. (1973). Angina pectoris at rest with preservation of exercise capacity: Prinzmetal’s variant angina. *Circulation*.

[52] Wilson R. F., Marcus M. L., White C. W. (1987). Prediction of the physiologic significance of coronary arterial lesions by quantitative lesion geometry in patients with limited coronary artery disease. *Circulation*.

[53] Harrison T. R., Reeves T. J., Harrison T. R., Reeves T. J. (1968). Some etiologic and epidemiologic considerations. *Principles and problems of ischemic heart disease*.

[54] Blumgart H. L., Schlesinger M. J., Davis D. (1940). Studies on the relation of the clinical manifestations of angina pectoris, coronary thrombosis and myocardial infarction to the pathologic findings. *American Heart Journal*.

[55] Blumgart H. L., Schlesinger M. J., Zoll P. M. (1941). Angina pectoris, coronary failure and acute myocardial infarction. The role of coronary occlusions and collateral circulation. *Journal of the American Medical Association*.

[56] Zoll P. M., Wessler S., Blumgart H. L. (1951). Angina pectoris: a clinical and pathologic correlation. *The American Journal of Medicine*.

[57] Langsted A., Kamstrup P. R., Nordestgaard B. G. (2014). Lipoprotein(a): fasting and nonfasting levels, inflammation, and cardiovascular risk. *Atherosclerosis*.

[58] Lange L. A., Carlson C. S., Hindorff L. A. (2006). Association of polymorphisms in the CRP gene with circulating C-reactive protein levels and cardiovascular events. *JAMA*.

[59] Dursunoğlu D., Evrengül H., Polat B. (2005). Lp(a) lipoprotein and lipids in patients with rheumatoid arthritis: serum levels and relationship to inflammation. *Rheumatology International*.

[60] Wang J., Hu B., Kong L., Cai H., Zhang C. (2008). Native, oxidized lipoprotein(a) and lipoprotein(a) immune complex in patients with active and inactive rheumatoid arthritis: plasma concentrations and relationship to inflammation. *Clinica Chimica Acta*.

[61] Zimmermann J., Herrlinger S., Pruy A., Metzger T., Wanner C. (1999). Inflammation enhances cardiovascular risk and mortality in hemodialysis patients. *Kidney International*.

[62] Oshunbade A. A., Kassahun-Yimer W., Valle K. A. (2021). Cigarette smoking, incident coronary heart disease, and coronary artery calcification in black adults: the Jackson Heart Study. *Journal of the American Heart Association*.

[63] Lagrand W. K., Visser C. A., Hermens W. T. (1999). C-reactive protein as a cardiovascular risk factor: more than an epiphenomenon?. *Circulation*.

[64] Montalescot G., Sechtem U., Achenbach S. (2013). ESC guidelines on the management of stable coronary artery disease: the Task Force on the management of stable coronary artery disease of the European Society of Cardiology. *European Heart Journal*.

[65] Ciliberti G., Guerra F., Coiro S., Capucci A. (2019). Is there an 'atherosclerotic continuum' from angina with unobstructed coronary arteries to MINOCA?. *European Heart Journal*.

[66] Miwa K., Fujita M., Sasayama S. (2005). Recent insights into the mechanisms, predisposing factors, and racial differences of coronary vasospasm. *Heart Vessels*.

[67] Yoshimura M., Yasue H., Nakayama M. (1998). A missense Glu 298Asp variant in the endothelial nitric oxide synthase gene is associated with coronary spasm in the Japanese. *Human Genetics*.

[68] Kaneda H., Taguchi J., Kuwada Y. (2006). Coronary artery spasm and the polymorphisms of the endothelial nitric oxide synthase gene. *Circulation Journal*.

[69] Ito T., Yasue H., Yoshimura M. (2002). Paraoxonase gene Gln192Arg (Q192R) polymorphism is associated with coronary artery spasm. *Human Genetics*.

[70] Scanlon P. J., Faxon D. P., Audet A. M. (1999). ACC/AHA guidelines for coronary angiography. A report of the American College of Cardiology/American Heart Association Task Force on practice guidelines (Committee on Coronary Angiography). Developed in collaboration with the Society for Cardiac Angiography and Interventions. *Journal of the American College of Cardiology*.

[71] Park J. S., Zhang S. Y., Jo S. H. (2006). Common adrenergic receptor polymorphisms as novel risk factors for vasospastic angina. *American Heart Journal*.

[72] Kaumann A. J., Levy F. O. (2006). 5-Hydroxytryptamine receptors in the human cardiovascular system. *Pharmacology & Therapeutics*.

[73] Oike Y., Hata A., Ogata Y., Numata Y., Shido K., Kondo K. (1995). Angiotensin converting enzyme as a genetic risk factor for coronary artery spasm. Implication in the pathogenesis of myocardial infarction. *Journal of Clinical Investigation*.

[74] Inoue N., Kawashima S., Kanazawa K., Yamada S., Akita H., Yokoyama M. (1998). Polymorphism of the NADH/NADPH oxidase p22 phox gene in patients with coronary artery disease. *Circulation*.

[75] Nakano T., Osanai T., Tomita H., Sekimata M., Homma Y., Okumura K. (2002). Enhanced activity of variant phospholipase C-delta1 protein (R257H) detected in patients with coronary artery spasm. *Circulation*.

[76] Murase Y., Yamada Y., Hirashiki A. (2004). Genetic risk and gene-environment interaction in coronary artery spasm in Japanese men and women. *European Heart Journal*.

[77] Horimoto M., Wakisaka A., Takenaka T. (1998). Familial evidence of vasospastic angina and possible involvement of HLA-DR2 in susceptibility to coronary spasm. *Japanese Circulation Journal*.

[78] Fournier J. A., Fernández-Cortacero J. A., Granado C., Gascón D. (1986). Familial migraine and coronary artery spasm in two siblings. *Clinical Cardiology*.

[79] Jackson M., Marks L., May G. H. W., Wilson J. B. (2018). The genetic basis of disease. *Essays in Biochemistry*.

[80] Lanza G. A., Careri G., Crea F. (2011). Mechanisms of coronary artery spasm. *Circulation*.

[81] Missala I., Kassner U., Steinhagen-Thiessen E. (2012). A systematic literature review of the association of lipoprotein(a) and autoimmune diseases and atherosclerosis. *International Journal of Rheumatology*.

[82] Koutroubakis I. E., Malliaraki N., Vardas E. (2001). Increased levels of lipoprotein (a) in Crohn’s disease: a relation to thrombosis?. *European Journal of Gastroenterology & Hepatology*.

[83] Maeda S., Abe A., Seishima M., Makino K., Noma A., Kawade M. (1989). Transient changes of serum lipoprotein(a) as an acute phase protein. *Atherosclerosis*.

[84] Holm S., Oma I., Hagve T.-A. (2019). Levels of Lipoprotein (a) in patients with coronary artery disease with and without inflammatory rheumatic disease: a cross-sectional study. *BMJ Open*.

[85] Müller N., Schulte D. M., Türk K. (2015). IL-6 blockade by monoclonal antibodies inhibits apolipoprotein (a) expression and lipoprotein (a) synthesis in humans. *Journal of Lipid Research*.

[86] Boffa M. B., Koschinsky M. L. (2016). Lipoprotein (a): truly a direct prothrombotic factor in cardiovascular disease?. *Journal of Lipid Research*.

[87] Hajjar K. A., Gavish D., Breslow J. L., Nachman R. L. (1989). Lipoprotein(a) modulation of endothelial cell surface fibrinolysis and its potential role in atherosclerosis. *Nature*.

[88] Rubanyi G. M., Freay A. D., Lawn R. M. (2000). Endothelium-dependent vasorelaxation in the aorta of transgenic mice expressing human apolipoprotein(a) or lipoprotein(a). *Endothelium*.

[89] Sorensen K. E., Celermajer D. S., Georgakopoulos D., Hatcher G., Betteridge D. J., Deanfield J. E. (1994). Impairment of endothelium-dependent dilation is an early event in children with familial hypercholesterolemia and is related to the lipoprotein(a) level. *Journal of Clinical Investigation*.

[90] Patthy L., Trexler M., Váli Z., Bányai L., Váradi A. (1984). Kringles: modules specialized for protein binding. Homology of the gelatin-binding region of fibronectin with the kringle structures of proteases. *FEBS Letters*.

[91] McCormick S. P. A., Schneider W. J. (2019). Lipoprotein(a) catabolism: a case of multiple receptors. *Pathology*.

[92] Kanaoka Y., Koga M., Sugiyama K., Ohishi K., Kataoka Y., Yamauchi A. (2017). Varenicline enhances oxidized LDL uptake by increasing expression of LOX-1 and CD36 scavenger receptors through *α*7 nAChR in macrophages. *Toxicology*.

[93] Schroeder J. S., Parmley W. W., Chatterjee K. (1991). Variant angina. *Cardiology*.

[94] Corcoran D., Young R., Adlam D. (2018). Coronary microvascular dysfunction in patients with stable coronary artery disease: the CE-MARC 2 coronary physiology sub-study. *International Journal of Cardiology*.

[95] JCS Joint Working Group (2014). Guidelines for diagnosis and treatment of patients with vasospastic angina (Coronary Spastic Angina) (JCS 2013). *Circulation Journal*.

[96] Sueda S., Kohno H., Fukuda H. (2004). Frequency of provoked coronary spasms in patients undergoing coronary arteriography using a spasm provocation test via intracoronary administration of ergonovine. *Angiology*.

[97] Bertrand M. E., LaBlanche J. M., Tilmant P. Y. (1982). Frequency of provoked coronary arterial spasm in 1089 consecutive patients undergoing coronary arteriography. *Circulation*.

[98] Sueda S., Kohno H., Fukuda H. (2004). Clinical impact of selective spasm provocation tests: comparisons between acetylcholine and ergonovine in 1508 examinations. *Coronary Artery Disease*.

[99] Suzuki Y., Tokunaga S., Ikeguchi S. (1992). Induction of coronary artery spasm by intracoronary acetylcholine: comparison with intracoronary ergonovine. *American Heart Journal*.

[100] Kanazawa K., Suematsu M., Ishida T. (1997). Disparity between serotonin- and acetylcholine-provoked coronary artery spasm. *Clinical Cardiology*.

[101] Sueda S., Kohno H., Fukuda H. (2003). Induction of coronary artery spasm by two pharmacological agents: comparison between intracoronary injection of acetylcholine and ergonovine. *Coronary Artery Disease*.

[102] Tada M., Kuzuya T., Inoue M. (1981). Elevation of thromboxane B2 levels in patients with classic and variant angina pectoris. *Circulation*.

[103] Okumura K., Yasue H., Horio Y. (1988). Multivessel coronary spasm in patients with variant angina: a study with intracoronary injection of acetylcholine. *Circulation*.

[104] Om S. Y., Yoo S. Y., Cho G. Y. (2020). Diagnostic and prognostic value of ergonovine echocardiography for noninvasive diagnosis of coronary vasospasm. *JACC Cardiovasc Imaging*.

[105] Yasue H., Nakagawa H., Itoh T., Harada E., Mizuno Y. (2008). Coronary artery spasm—clinical features, diagnosis, pathogenesis, and treatment. *Journal of Cardiology*.

[106] Nakayama M., Yasue H., Yoshimura M. (1999). T-786-->C mutation in the 5′-flanking region of the endothelial nitric oxide synthase gene is associated with coronary spasm. *Circulation*.

[107] Okumura K., Yasue H., Matsuyama K. (1996). Diffuse disorder of coronary artery vasomotility in patients with coronary spastic angina. Hyperreactivity to the constrictor effects of acetylcholine and the dilator effects of nitroglycerin. *Journal of the American College of Cardiology*.

[108] Schlaich M. P., John S., Langenfeld M. R., Lackner K. J., Schmitz G., Schmieder R. E. (1998). Does lipoprotein(a) impair endothelial function?. *Journal of the American College of Cardiology*.

[109] Moeslinger T., Friedl R., Volf I., Brunner M., Koller E., Spieckermann P. G. (2000). Inhibition of inducible nitric oxide synthesis by oxidized lipoprotein(a) in a murine macrophage cell line. *FEBS Letters*.

[110] Weinberg J. B., Misukonis M. A., Shami P. J. (1995). Human mononuclear phagocyte inducible nitric oxide synthase (iNOS): analysis of iNOS mRNA, iNOS protein, biopterin, and nitric oxide production by blood monocytes and peritoneal macrophages. *Blood*.

[111] MacMicking J., Xie Q. W., Nathan C. (1997). Nitric oxide and macrophage function. *Annual Review of Immunology*.

[112] Niedbala W., Cai B., Liew F. Y. (2006). Role of nitric oxide in the regulation of T cell functions. *Annals of the rheumatic diseases*.

[113] Niedbala W., Alves-Filho J. C., Fukada S. Y. (2011). Regulation of type 17 helper T-cell function by nitric oxide during inflammation. *Proceedings of the National Academy of Sciences of the United States of America*.

[114] Gunnett C. A., Heistad D. D., Loihl A., Faraci F. M. (2000). Tumor necrosis factor-alpha impairs contraction but not relaxation in carotid arteries from iNOS-deficient mice. *The American Journal of Physiology-Regulatory, Integrative and Comparative Physiology*.

[115] Gunnett C. A., Lund D. D., McDowell A. K., Faraci F. M., Heistad D. D. (2005). Mechanisms of inducible nitric oxide synthase-mediated vascular dysfunction. *Arteriosclerosis, Thrombosis, and Vascular Biology*.

[116] Fukumoto Y., Shimokawa H., Kozai T. (1997). Vasculoprotective role of inducible nitric oxide synthase at inflammatory coronary lesions induced by chronic treatment with interleukin-1beta in pigs in vivo. *Circulation*.

[117] Sica A., Mantovani A. (2012). Macrophage plasticity and polarization: in vivo veritas. *Journal of Clinical Investigation*.

[118] Umemoto S., Suzuki N., Fujii K. (2000). Eosinophil counts and plasma fibrinogen in patients with vasospastic angina pectoris. *The American Journal of Cardiology*.

[119] Hung M. J., Cherng W. J., Cheng C. W., Li L. F. (2006). Comparison of serum levels of inflammatory markers in patients with coronary vasospasm without significant fixed coronary artery disease versus patients with stable angina pectoris and acute coronary syndromes with significant fixed coronary artery disease. *The American Journal of Cardiology*.

[120] Yun K. H., Oh S. K., Park E. M. (2006). An increased monocyte count predicts coronary artery spasm in patients with resting chest pain and insignificant coronary artery stenosis. *The Korean Journal of Internal Medicine*.

[121] Hung M. J., Chang N. C., Hu P. (2021). Association between coronary artery spasm and the risk of incident diabetes: a nationwide population-based cohort study. *International Journal of Medical Sciences*.

[122] Shah N. H., Schneider T. R., DeFaria Y. D., Cahill K. N., Laidlaw T. M. (2016). Eosinophilia-associated coronary artery vasospasm in patients with aspirin-exacerbated respiratory disease. *The Journal of Allergy and Clinical Immunology*.

[123] Minai-Fleminger Y., Levi-Schaffer F. (2009). Mast cells and eosinophils: the two key effector cells in allergic inflammation. *Inflammation Research*.

[124] Scotton C. J., Martinez F. O., Smelt M. J. (2005). Transcriptional profiling reveals complex regulation of the monocyte IL-1 beta system by IL-13. *Journal of Immunology*.

[125] Chinetti-Gbaguidi G., Colin S., Staels B. (2015). Macrophage subsets in atherosclerosis. *Nature Reviews Cardiology*.

[126] Alternative activation of macrophages: an immunologic functional perspective. https://pubmed.ncbi.nlm.nih.gov/19105661/.

[127] van der Valk F. M., Bekkering S., Kroon J. (2016). Oxidized phospholipids on lipoprotein(a) elicit arterial wall inflammation and an inflammatory monocyte response in humans. *Circulation*.

[128] Aicher A., Heeschen C., Mohaupt M., Cooke J. P., Zeiher A. M., Dimmeler S. (2003). Nicotine strongly activates dendritic cell-mediated adaptive immunity: potential role for progression of atherosclerotic lesions. *Circulation*.

[129] Mantovani A., Sica A., Locati M. (2005). Macrophage polarization comes of age. *Immunity*.

[130] Yang X., Galeano N. F., Szabolcs M., Sciacca R. R., Cannon P. J. (1996). Oxidized low density lipoproteins alter macrophage lipid uptake, apoptosis, viability and nitric oxide synthesis. *Journal of Nutrition*.

[131] Lanza G. A., Pedrotti P., Pasceri V., Lucente M., Crea F., Maseri A. (1996). Autonomic changes associated with spontaneous coronary spasm in patients with variant angina. *Journal of the American College of Cardiology*.

[132] Lee R. H., Vazquez G. (2013). Evidence for a prosurvival role of alpha-7 nicotinic acetylcholine receptor in alternatively (M2)-activated macrophages. *Physiological Reports*.

[133] Lu C. H., Lai C. Y., Yeh D. W. (2018). Involvement of M1 macrophage polarization in endosomal toll-like receptors activated psoriatic inflammation. *Mediators of Inflammation*.

[134] Kamio Y., Miyamoto T., Kimura T. (2018). Roles of nicotine in the development of intracranial aneurysm rupture. *Stroke*.

[135] Libby P. (2002). Inflammation in atherosclerosis. *Nature*.

[136] Borovikova L. V., Ivanova S., Zhang M. (2000). Vagus nerve stimulation attenuates the systemic inflammatory response to endotoxin. *Nature*.

[137] Newhouse P., Kellar K., Aisen P. (2012). Nicotine treatment of mild cognitive impairment: a 6-month double-blind pilot clinical trial. *Neurology*.

[138] Riches K., Franklin L., Maqbool A. (2013). Apolipoprotein(a) acts as a chemorepellent to human vascular smooth muscle cells via integrin *α*V*β*3 and RhoA/ROCK-mediated mechanisms. *The International Journal of Biochemistry & Cell Biology*.

[139] Ichikawa T., Unoki H., Sun H. (2002). Lipoprotein(a) promotes smooth muscle cell proliferation and dedifferentiation in atherosclerotic lesions of human apo(a) transgenic rabbits. *The American Journal of Pathology*.

[140] Wang Y., Zheng X. R., Riddick N. (2009). ROCK isoform regulation of myosin phosphatase and contractility in vascular smooth muscle cells. *Circulation Research*.

[141] Ridker P. M., Everett B. M., Thuren T. (2017). Antiinflammatory therapy with canakinumab for atherosclerotic disease. *The New England Journal of Medicine*.

[142] Tardif J.-C., Kouz S., Waters D. D. (2019). Efficacy and safety of low-dose colchicine after myocardial infarction. *The New England Journal of Medicine*.

[143] Ridker P. M., Everett B. M., Pradhan A. (2019). Low-dose methotrexate for the prevention of atherosclerotic events. *The New England Journal of Medicine*.

[144] Schultz O., Oberhauser F., Saech J. (2010). Effects of inhibition of interleukin-6 signalling on insulin sensitivity and lipoprotein (a) levels in human subjects with rheumatoid diseases. *PLoS One*.

[145] Galitovskiy V., Qian J., Chernyavsky A. I. (2011). Cytokine-induced alterations of *α*7 nicotinic receptor in colonic CD4 T cells mediate dichotomous response to nicotine in murine models of Th1/Th17- versus Th2-mediated colitis. *Journal of Immunology*.

[146] Burgess S., Ference B. A., Staley J. R. (2018). European Prospective Investigation Into Cancer and Nutrition–Cardiovascular Disease (EPIC-CVD) Consortium, Association of LPA variants with risk of coronary disease and the implications for lipoprotein(a)-lowering therapies: a Mendelian randomization analysis. *JAMA Cardiology*.

[147] Lamina C., Kronenberg F. (2019). Lp(a)-GWAS-Consortium, Estimation of the required lipoprotein(a)-lowering therapeutic effect size for reduction in coronary heart disease outcomes: a Mendelian randomization analysis. *JAMA Cardiology*.

[148] Pepine C. J., Geller N. L., Knatterud G. L. (1994). The Asymptomatic Cardiac Ischemia Pilot (ACIP) study: design of a randomized clinical trial, baseline data and implications for a long-term outcome trial. *Journal of the American College of Cardiology*.

[149] Gibbons R. J., Abrams J., Chatterjee K. (2003). ACC/AHA 2002 guideline update for the management of patients with chronic stable angina--summary article: a report of the American College of Cardiology/American Heart Association Task Force on practice guidelines (Committee on the Management of Patients With Chronic Stable Angina). *Journal of the American College of Cardiology*.

[150] Yasue H., Omote S., Takizawa A. (1981). Comparison of coronary arteriographic findings during angina pectoris associated with S-T elevation or depression. *The American Journal of Cardiology*.

[151] Cheng T. O. (2007). Ergonovine test for coronary artery spasm. *International Journal of Cardiology*.

[152] Gibbons R. J., Chatterjee K., Daley J. (1999). ACC/AHA/ACP-ASIM guidelines for the management of patients with chronic stable angina: a report of the American College of Cardiology/American Heart Association Task Force on Practice Guidelines (Committee on Management of Patients With Chronic Stable Angina). *Journal of the American College of Cardiology*.

[153] Hung M. J., Hu P., Hung M. Y. (2014). Coronary artery spasm: review and update. *International Journal of Medical Sciences*.

[154] Beltrame J. F., Sasayama S., Maseri A. (1999). Racial heterogeneity in coronary artery vasomotor reactivity: differences between Japanese and Caucasian patients. *Journal of the American College of Cardiology*.

[155] Kawana A., Takahashi J., Takagi Y. (2013). Gender differences in the clinical characteristics and outcomes of patients with vasospastic angina--a report from the Japanese Coronary Spasm Association. *Circulation Journal*.

[156] Saw J., Aymong E., Mancini G. B., Sedlak T., Starovoytov A., Ricci D. (2014). Nonatherosclerotic coronary artery disease in young women. *Canadian Journal of Cardiology*.

[157] Ong P., Athanasiadis A., Hill S., Vogelsberg H., Voehringer M., Sechtem U. (2008). Coronary artery spasm as a frequent cause of acute coronary syndrome: the CASPAR (Coronary Artery Spasm in Patients With Acute Coronary Syndrome) Study. *Journal of the American College of Cardiology*.

[158] Truskey G. A. (2010). Endothelial cell vascular smooth muscle cell co-culture assay for high throughput screening assays for discovery of anti-angiogenesis agents and other therapeutic molecules. *International Journal of High Throughput Screening*.

[159] Davies P. F. (1995). Flow-mediated endothelial mechanotransduction. *Physiological Reviews*.

[160] Passerini A. G., Polacek D. C., Shi C. (2004). Coexisting proinflammatory and antioxidative endothelial transcription profiles in a disturbed flow region of the adult porcine aorta. *Proceedings of the National Academy of Sciences of the United States of America*.

[161] Tsai M. C., Chen L., Zhou J. (2009). Shear stress induces synthetic-to-contractile phenotypic modulation in smooth muscle cells via peroxisome proliferator-activated receptor alpha/delta activations by prostacyclin released by sheared endothelial cells. *Circulation Research*.

[162] Iyer R. P., de Castro Brás L. E., Jin Y. F., Lindsey M. L. (2014). Translating Koch’s postulates to identify matrix metalloproteinase roles in postmyocardial infarction remodeling: cardiac metalloproteinase actions (CarMA) postulates. *Circulation Research*.

[163] Hung M. Y., Hsu K. H., Hung M. J., Cheng C. W., Kuo L. T., Cherng W. J. (2009). Interaction between cigarette smoking and high-sensitivity C-reactive protein in the development of coronary vasospasm in patients without hemodynamically significant coronary artery disease. *The American Journal of the Medical Sciences.*.

[164] Yeang C., Cotter B., Tsimikas S. (2016). Experimental animal models evaluating the causal role of lipoprotein(a) in atherosclerosis and aortic stenosis. *Cardiovascular Drugs and Therapy*.

[165] Scirica B. M., Morrow D. A. (2006). Is C-reactive protein an innocent bystander or proatherogenic culprit? The verdict is still out. *Circulation*.

[166] Jialal I., Devaraj S., Venugopal S. K. (2004). C-reactive protein: risk marker or mediator in atherothrombosis?. *Hypertension*.

[167] Ridker P. M. (2007). C-reactive protein and the prediction of cardiovascular events among those at intermediate risk: moving an inflammatory hypothesis toward consensus. *Journal of the American College of Cardiology*.

[168] Bisoendial R. J., Kastelein J. J., Levels J. H. (2005). Activation of inflammation and coagulation after infusion of C-reactive protein in humans. *Circulation Research*.

[169] Danenberg H. D., Szalai A. J., Swaminathan R. V. (2003). Increased thrombosis after arterial injury in human C-reactive protein-transgenic mice. *Circulation*.

[170] Jialal I., Vikram N. K. (2018). Inflammation and atherosclerosis: fulfilling Koch’s postulates. *Therapeutic Advances in Cardiovascular Disease*.

[171] Doo Y. C., Kim D. M., Oh D. J., Ryu K. H., Rhim C. Y., Lee Y. (2001). Effect of beta blockers on expression of interleukin-6 and C-reactive protein in patients with unstable angina pectoris. *The American Journal of Cardiology*.

[172] Loppnow H., Zhang L., Buerke M. (2011). Statins potently reduce the cytokine-mediated IL-6 release in SMC/MNC cocultures. *Journal of Cellular and Molecular Medicine*.

[173] Matsumori A., Nishio R., Nose Y. (2010). Calcium channel blockers differentially modulate cytokine production by peripheral blood mononuclear cells. *Circulation Journal*.

[174] Sindler A. L., Fleenor B. S., Calvert J. W. (2011). Nitrite supplementation reverses vascular endothelial dysfunction and large elastic artery stiffness with aging. *Aging Cell*.

