# Supplementary material for: Apolipoprotein (a)/Lipoprotein(a)-Induced Oxidative-Inflammatory α7-nAChR/p38 MAPK/IL-6/RhoA-GTP Signaling Axis and M1 Macrophage Polarization Modulate Inflammation-Associated Development of Coronary Artery Spasm
Source: Oxid Med Cell Longev. 2022 Jan 19;2022:9964689. doi: 10.1155/2022/9964689 (PMC8793348; doi:10.1155/2022/9964689)
Supplement: Supplementary 1 — Supplementary Table S1: western blot antibody sheet. [file 9964689.f1.pdf]

Supplementary Table S1. Western blot antibodies sheet.

No.	Target	Dilution	Catalog		Kda
1	α7nAChR	1:1000	abcam	ab216485	47
2	p38	1:1000	abcam	ab31828	37
3	p-pp38	1:1000	abcam	ab4822	37
4	IL-6	1:1000	abcam	ab9324	120
5	RhoA	1:1000	cellsignal	#2117	25
6	RhoA-GTP	1:1000	cellsignal	#8820	25
7	p-MBS	1:1000	thermofisher	#3040	140
8	t-MBS	1:1000	thermofisher	#2634	140
9	ROCK1	1:1000	cellsignal	#4035	160
10	ROCK2	1:1000	cellsignal	#9029	160
11	iNOS	1:1000	abcam	ab3523	131
12	GAPDH	1:10000	abcam	ab9484	37
