# Supplementary figures and images for: Apolipoprotein (a)/Lipoprotein(a)-Induced Oxidative-Inflammatory α7-nAChR/p38 MAPK/IL-6/RhoA-GTP Signaling Axis and M1 Macrophage Polarization Modulate Inflammation-Associated Development of Coronary Artery Spasm
Source: Oxid Med Cell Longev. 2022 Jan 19;2022:9964689. doi: 10.1155/2022/9964689 (PMC8793348; doi:10.1155/2022/9964689)

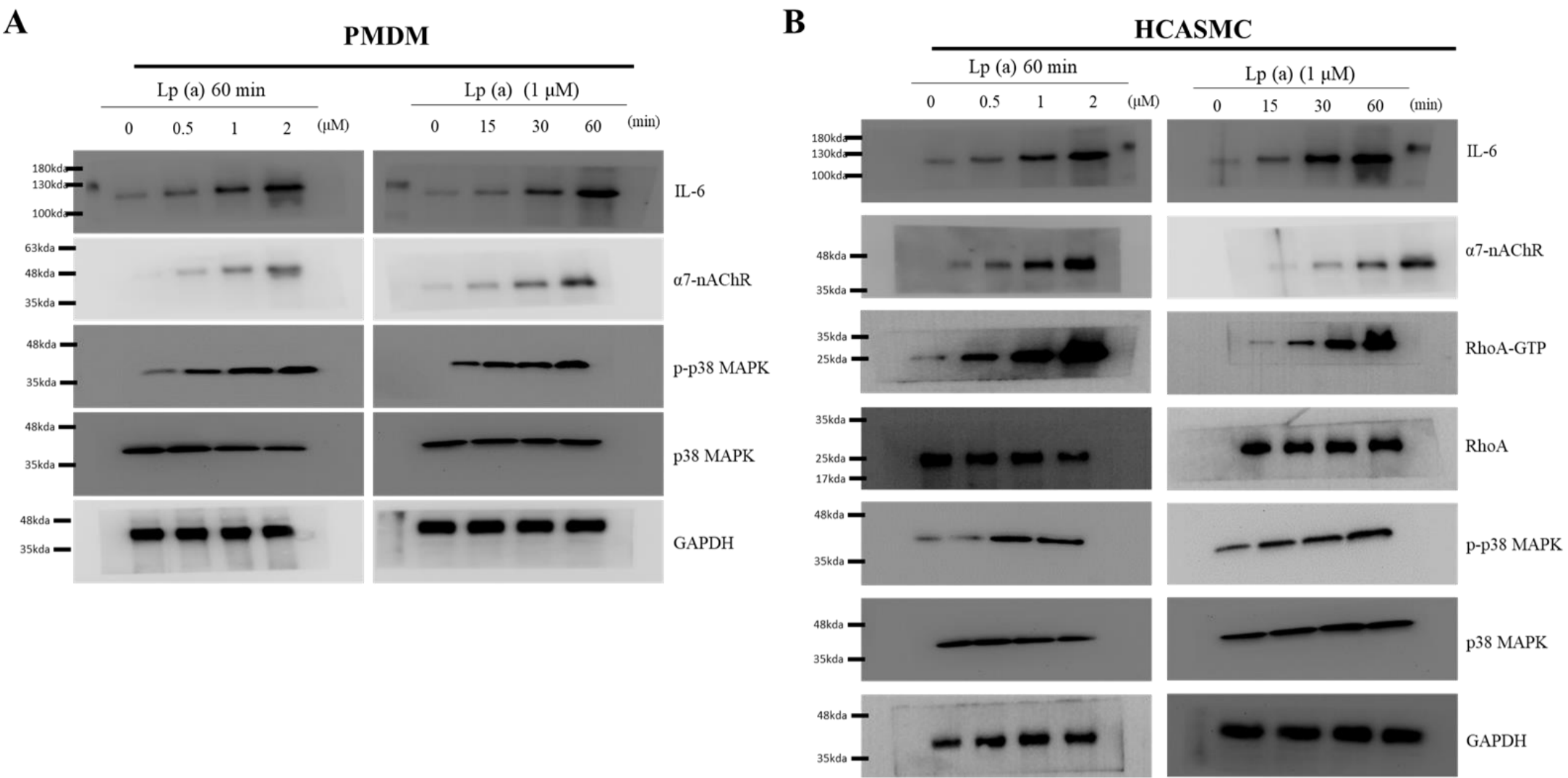

**Supplementary Figure S1. Full-size blots of Figure 2 (a) and 2(b).**

Supplement: Supplementary 2 — Supplementary Figure S1: full-size blots of Figures 2(a) and 2(b). [file 9964689.f2.pdf]

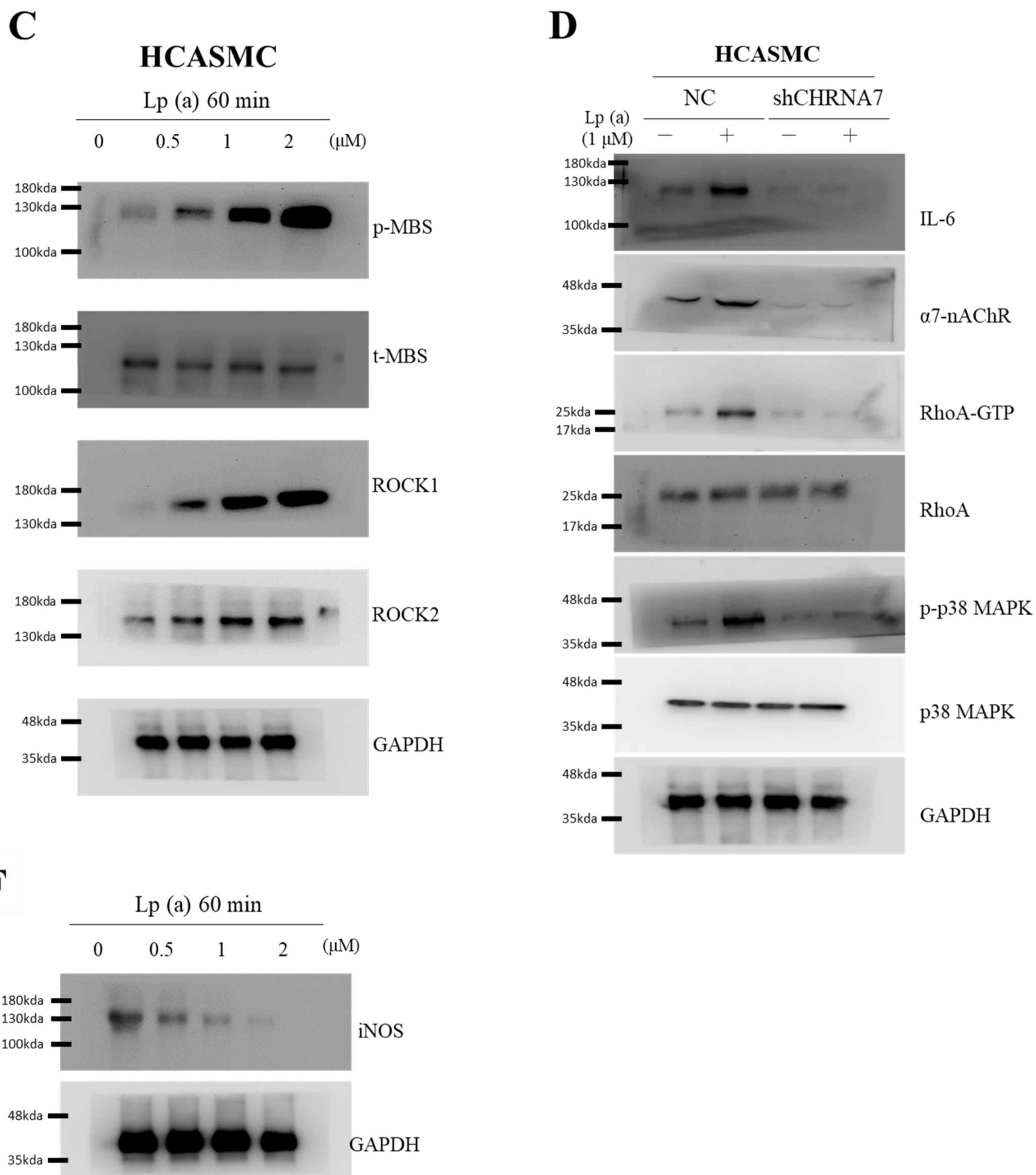

**Supplementary Figure S2. Full-size blots of Figure 2 (c), 2 (d) and 2(f).**

Supplement: Supplementary 3 — Supplementary Figure S2: full-size blots of Figures 2(c), 2(d), and 2(f). [file 9964689.f3.pdf]

C

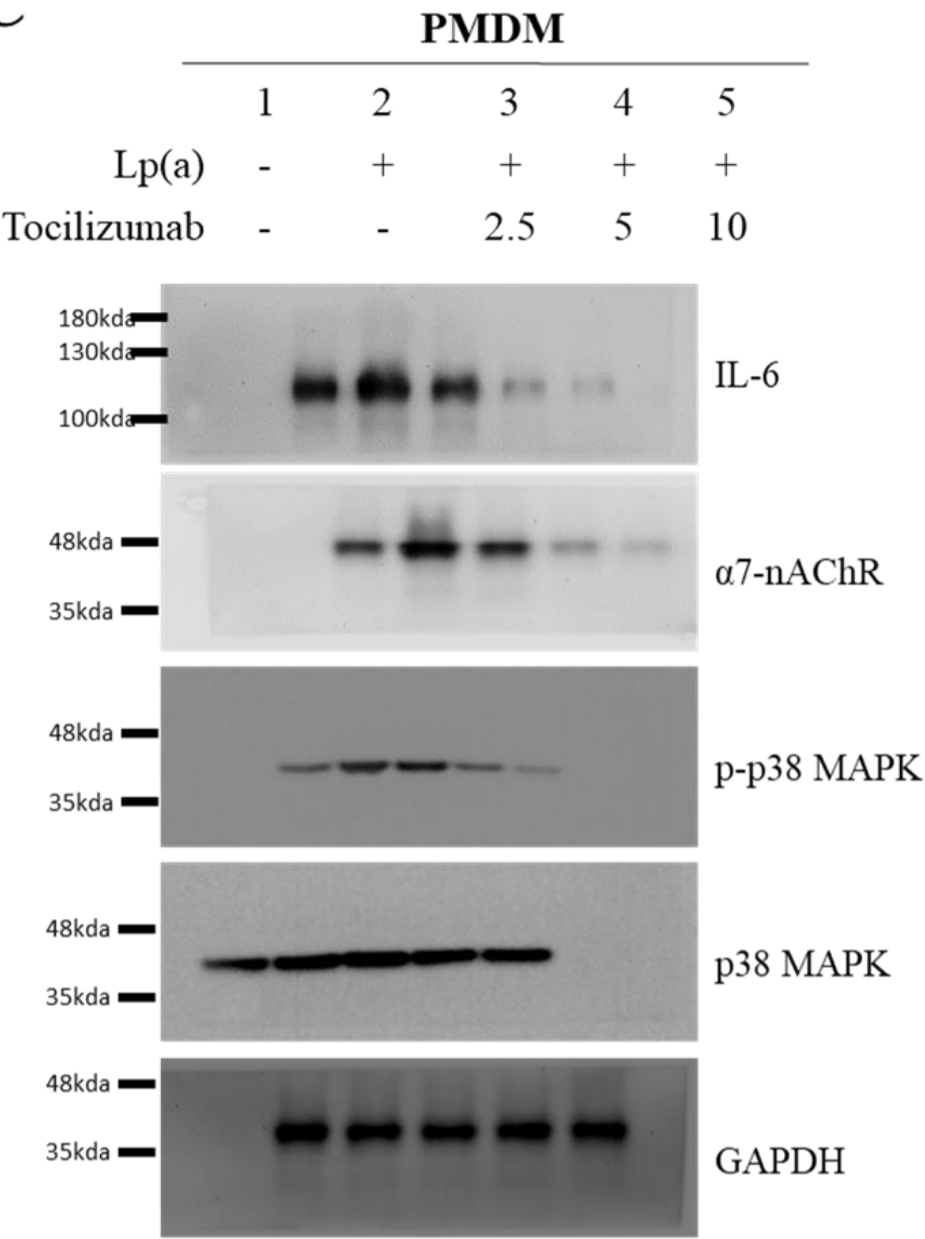

D

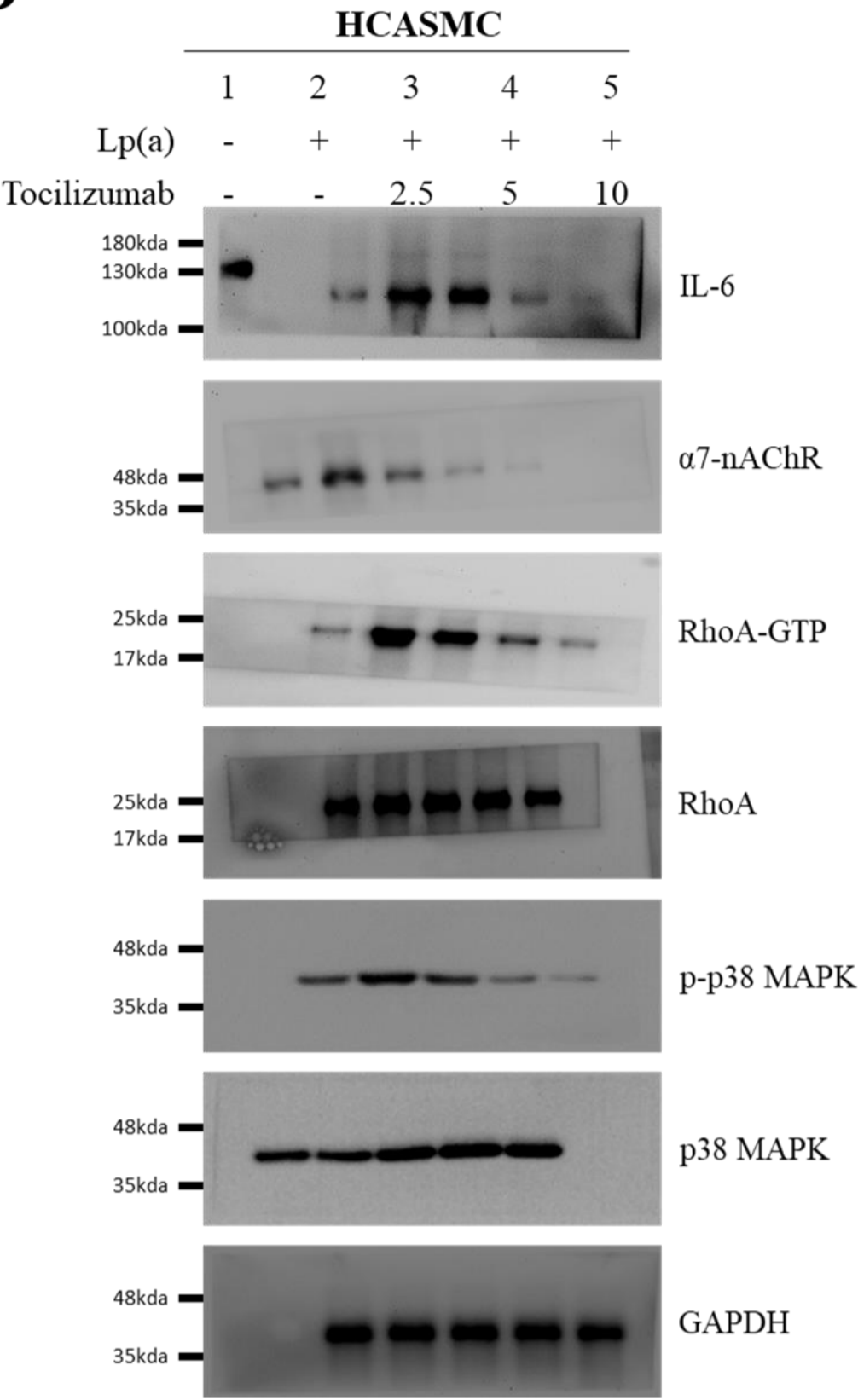

Supplementary Figure S3. Full-size blots of Figure 4 (c) and 4 (d).

Supplement: Supplementary 4 — Supplementary Figure S3: full-size blots of Figures 4(c) and 4(d). [file 9964689.f4.pdf]
